# Risk Factors for Cardiovascular Disease: Epidemiology, Screening, Prevention, and Therapeutic Interventions

**DOI:** 10.1002/mco2.70869

**Published:** 2026-07-17

**Authors:** Mowei Kong, Zaiyong Zheng, Min Huang, Shengyu Huang, Xingwei Zhao, Yang Yu, Chunxiang Zhang

**Affiliations:** ^1^ Department of Cardiology The Affiliated Hospital of Southwest Medical University Luzhou Sichuan Province China; ^2^ Department of Cardiology Panzhihua Central Hospital Panzhihua Sichuan China; ^3^ Key Laboratory of Medical Electrophysiology Institute of Cardiovascular Research Ministry of Education Luzhou Sichuan Province China; ^4^ Basic Medicine Research Innovation Center For Cardiometabolic Diseases Ministry of Education Southwest Medical University Luzhou Sichuan Province China; ^5^ Nucleic Acid Medicine of Luzhou Key Laboratory Luzhou Sichuan China

**Keywords:** air pollution, cardiovascular disease (CVD), emerging risk factors, genetics and epigenetics, prevention and therapeutic interventions, risk factors, risk screening and assessment, traditional risk factors

## Abstract

Cardiovascular diseases (CVDs) remain the leading cause of morbidity and mortality worldwide, and the burden is particularly severe in low‐income and middle‐income countries. Although traditional risk factors such as hypertension, dyslipidemia, diabetes, and smoking have been well established, new research evidence indicates that inflammation, environmental exposure, psychological and social stress, gut microbiota, and genetic susceptibility play a crucial role in shaping the risk profile of CVDs. This review comprehensively summarizes the epidemiology, pathophysiological mechanisms, screening strategies, prevention, and treatment interventions of CVDs throughout the disease development process. Special emphasis is placed on the integration of multiomics methods, artificial intelligence (AI), and digital health technologies (including wearable devices and AI‐enhanced electrocardiograms), which are transforming risk prediction and personalized prevention approaches. We also summarize current preclinical and clinical evidence, including ongoing trials, and discuss implementation differences in different socioeconomic environments. Despite significant progress, there are still many challenges in translating new biomarkers and technologies into scalable and equitable clinical applications. This review identifies key knowledge gaps and proposes future directions toward precision cardiovascular medicine, aiming to combine mechanistic insights with practical applications, ultimately reducing the burden of CVDs globally.

## Graphical abstract

Cardiovascular disease arises from lifelong accumulation of traditional and emerging risk factors that converge on shared pathophysiological pathways, leading to diverse clinical outcomes, and can be mitigated through integrated screening, prevention, and precision therapies.

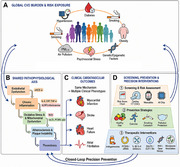

## 1 Introduction

Cardiovascular disease (CVD) is still the leading cause of global mortality in 2023, with 19.2 million deaths, up from 13.1 million in 1990. At present, ischemic heart disease and stroke account for four‐fifths of all CVD deaths worldwide. More than 75% of those deaths occur in low‐ and middle‐income countries (LMICs), which have the highest incidence and worst affordability.

The occurrence of CVD highly relies on risk factors, and epidemiology results highlight that controlling risk factors can significantly reduce the incidence of CVD. Approximately 90% of the population‐attributed risk of acute myocardial infarction (MI) worldwide is attributed to measurable and modifiable risk factors [1]. In addition, 70% of CVD cases and deaths are attributed to modifiable risk factors [2]. Moreover, owing to the limited regeneration capability of the myocardium, any injury to the heart may lead to the permanent loss of cardiomyocytes. Therefore, long‐term and different risk factors have a cumulative effect on cardiac injury, which may start at a very young age [3]. Finally, most acute CVD events are fatal, and approximately 60% of sudden deaths are related to CVD [4]. Therefore, controlling cardiovascular risk factors offers substantial preventive value and represents a highly cost‐effective strategy for reducing the burden of CVD. Traditional risk factors, such as high arterial pressure, elevated cholesterol and glucose levels, and cigarette smoking, are well recognized. The prevention of these factors has been widely implemented in most countries, and the effects have become apparent [5, 6, 7, 8].

**FIGURE 1:**
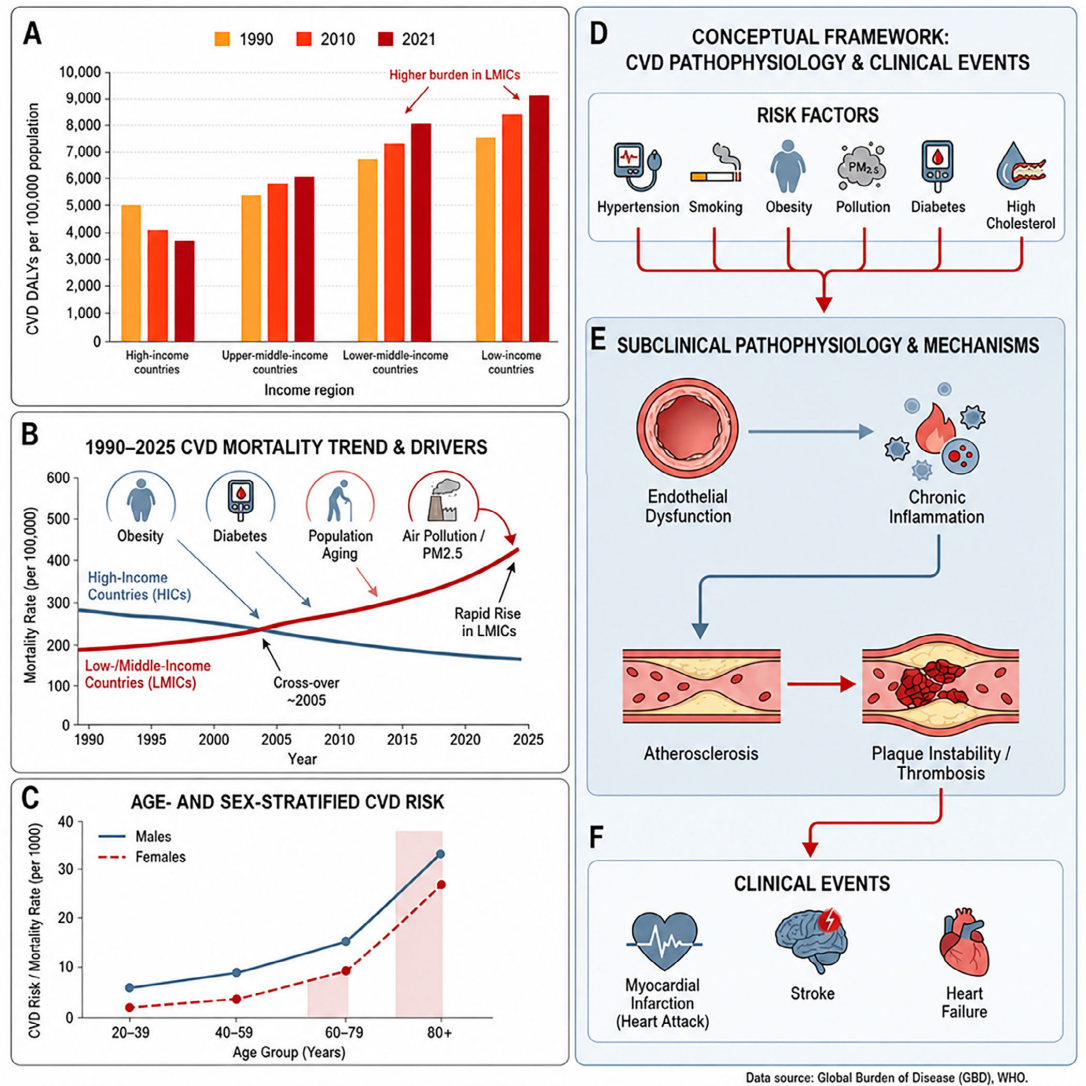
Global burden of cardiovascular disease and an integrated framework linking risk factors to clinical events. (Draft diagram created in WPS office PPT with GPT 5.5‐assisted layout suggestions. Final image refined and annotated by the authors. All pathway details were cross‐checked against experimental data.) (A) Global distribution of CVD burden—Regional burden of cardiovascular disease measured by disability‐adjusted life years (DALYs). (B) Mortality trends by income level—Trends in CVD mortality across countries with different income levels from 1990 to 2025. (C) Impact of obesity—Contribution of obesity to the global burden of cardiovascular disease. (D) Impact of diabetes—Contribution of diabetes to cardiovascular disease risk and burden. (E) Impact of population aging—Age‐related changes in cardiovascular disease burden and risk. (F) Impact of PM2.5 exposure—Cardiovascular burden attributable to ambient particulate air pollution.

Although intensive control of traditional risk factors has led to substantial reductions in CVD incidence, a considerable burden of CVD persists among treated individuals. Large clinical trials and contemporary meta‐analyses consistently demonstrate substantial residual cardiovascular risk even when traditional risk factors are controlled [9, 10]. For example, after high‐intensity statin therapy, 38% of adverse events are associated with a risk of being free of clinical atherosclerotic CVD patients [11]. In patients with previous MI, after the use of aggressive secondary prevention strategies, anti‐inflammatory therapy can still lower the risk of adverse cardiovascular events by 15%. These results indicate that unrevealed emerging risk factors play a vital role in CVD risk after traditional risk factors have been controlled [12]. Recent epidemiology studies revealed that air pollution, social determinants, and unhealthy lifestyles also contribute to CVD. Epidemiologic studies have revealed that CVD mortality risk will increase by 8–18% per 10 µg/m^3^ increase in fine particles due to air pollution [13]. More importantly, certain emerging risk factors may enhance the intergenerational inheritance of CVD susceptibility (Figure 1). However, the underlying mechanism is still unknown.

Therefore, this work aims to provide a comprehensive and in‐depth review of the traditional and emerging risk factors for CVD. Moreover, screening, prevention, and therapeutic interventions involving these factors are discussed.

## 2 Current Epidemiological Status of CVDs

The global CVD landscape is sharply polarized: HICs face slowing mortality decline but rising chronic disability, whereas LMICs continue to experience increasing mortality dominated by acute, early‐onset events, necessitating stage‐specific and region‐tailored prevention and care strategies.

### 2.1 Global and Regional Distributions

The global disease burden has shifted from infectious to noncommunicable diseases, with CVD now leading. However, while high‐income countries (HICs) see falling CVD deaths through prevention and care, middle‐income countries face a surge of cases as risks outpace health‐system adaptation, and low‐income countries confront a triple threat of persistent infections, poverty‐related heart conditions, and rising CVD risk factors [14]. Global health trends have undergone significant transformations over the past several decades, driven by various factors, such as advancements in medical technology and health systems and lifestyle changes [15]. In HICs, there has been a steady reduction in CVD mortality, owing to improvements in the prevention and treatment of CVD risk factors such as smoking, high blood pressure, and high cholesterol [16]. These countries have also seen rapid advancements in medical interventions and technologies, leading to better survival rates from acute conditions such as heart attacks [17]. However, as CVD survival improves, new challenges such as chronic heart failure (HF) and disability from CVD are emerging as significant health burdens [18].

In contrast, the epidemiological landscape in LMICs presents a different story [19]. While these regions are experiencing an increasing burden of CVD, the trends are not uniform [20]. In low‐income countries, particularly in Sub‐Saharan Africa, CVD continues to cause high mortality rates, often in the form of acute events [21]. These nations still struggle with conditions such as rheumatic heart disease, which remain prevalent and affect younger populations [22]. On the other hand, middle‐income countries, such as China, are witnessing a rapid increase in CVD deaths due to lifestyle changes and rising rates of hypertension, smoking, and diabetes [23].

HICs have already started their “epidemiologic pivot,” marked by a 40–60% reduction in age‐standardized CVD deaths (United States has declined by 53%, and the United Kingdom has dropped by 46%) in the last four decades, owing mainly to a population‐level decrease in smoking from approximately 40% in the early 1970s to <15% in the present, better control of blood pressure and lipid levels, and a more healthy diet [24]. Emergency medical and interventional technologies are also rapidly advancing [25]. In cardiology, fewer people die of acute MI in the hospital [26]. In the United States, this figure is only 5–7% [27]. However, these successes have dramatically reshaped the picture: there are more than 6.5 million adult Americans living with HF today, and this figure is expected to rise to 9 million by 2030 [28]. In addition, more than 9% of adults over 65 years old suffer from AF [29]. The gains from survival have been quickly eroded away by high rates of recurrent hospitalization (over 25% within 1 year of HF diagnosis) and ongoing increases in DALYs from chronic CVD [30]. In 2023, multiple HICs reported continued downward trends in CVD mortality but increasing CVD‐related DALYs and an increasing need for a shift away from “preventing death” toward “preventing disability” and improving overall quality of life [31].

In poor countries, especially in sub‐Saharan Africa, CVD continues to occur as an acute condition with high mortality, with CVD being responsible for more deaths annually than all other types of cancer combined [32]. GBD 2021 shows a higher age‐standardized CVD death rate of 350–400 per 100,000 compared with less than 150 in high‐income regions [33]. In‐hospital AMI mortality in poor countries exceeds 20%, being three to four times higher than the rates reported in Europe and North America [34]. Rheumatic heart disease remains a major cause of burden worldwide [35], affecting more than 39 million individuals (over 70% of the global burden, or >10 times that of HICs), and is the leading cause of HF among the younger population. The median age of onset for HF was ∼50 years, earlier than that in Western cohorts; the 2‐year survival rate was <50% [36]. Hypertension control was less than 20%, and stroke mortality was two to three times as high as that in wealthier nations [37]. Given the “early‐onset, high‐fatality” profile, investments in health systems, emergency care capacity, and population‐wide prevention of all causes of stroke are critical [38].

Middle‐income countries now constitute the new epicenter of the global CVD burden, with China exemplifying this trend [38]. As shown by the China Cardiovascular Health & Disease Report 2024, CVD mortality has increased since the 1990s [39]. Every year, more than 4 million Chinese people die (approximately 43% of total deaths), with CVD at the top of the causes of death [40]. Approximately 68% of these deaths are associated with modifiable factors: hypertension (population‐attributable fraction: 24%), smoking (18%), and ambient air pollution (PM2.5 and NO_2_, 13%) [41]. The prevalence of adult hypertension in China was 27.5%, but only 15% were controlled [42]. Diabetes affects more than 12% of adults, with half undiagnosed, and the combined overweight and obesity rate is >34% and continues to increase [43]. As such, it results in two pressures: one on managing acute events and the other on a continuously growing chronic disease pool [44].

The fact that there is so much variation across Chinese subgroups further complicates the task of homogenization [45]. China's urban AMI in‐hospital mortality rate is between 6 and 8%, which is on par with Western data, whereas rural rates remain greater than 15% [46]. Stroke mortality is more than 40% higher in rural Chinese villages than in city villages in the central and western provinces [47]. By sex, men aged 35–55 years have a coronary disease incidence rate two to three times greater than that of women, with a male smoking prevalence of 45 versus 3% in women [48]. After menopause, female risk accelerates and surpasses male risk beyond the age of 70 years [49]. Two‐thirds of China's CVD deaths occur among adults ≥65 years, yet stroke in the 40–60 years age group accounts for ∼30% of national stroke DALYs—far above the share seen in high‐income populations [50]. Effective CVD control in China therefore requires not only a unified national strategy but also region‐, sex‐, and life‐stage‐specific interventions that extend from primordial prevention to long‐term chronic disease management [51].

### 2.2 Temporal Trends

Over the past four decades, with the global epidemiologic landscape of CVD for the past four decades, the global landscape of CVD has substantially fragmented—the end results reveal two distinct faces of the coin for public health/medical successes and failures while simultaneously revealing evidence of the marked influence of urbanization, globalization, and environmental forces on population health [52].

HICs started to see gains in the 1970s through aggressive tobacco control (which caused the smoking rate to fall from approximately 40% to under 15%), optimal management of HTN and hyperlipidemia, and a move toward healthier diets—which also led to a significant decline in age‐standardized CVD mortality—United States: 53%, United Kingdom: 46% reduction between 1975 and 2019 [53]. In‐hospital acute MI fatality, however, increased over this period [54]. The incidence of HF has decreased from >20% to 5–7% [55]. However, the new century has led to marked deceleration [56]. Obesity (>40% of US adults) and diabetes (∼11%) have erased some of these gains [57]. More importantly, the disease spectrum has shifted: the United States now has 6.5 million HF patients, a figure projected to reach 9 million by 2030, and atrial fibrillation (AF) affects >9% of people ≥65 years [58]. Although total CVD‐related DALYs continue to decrease, the number of years lived with disability from HF has increased almost 40% since 2000 [59]. The strategic focus in HICs is therefore moving from “reducing mortality” to “delaying progression and protecting quality of life,” demanding stronger long‐term care and rehabilitation systems [60].

Unlike HICs, where the burden decreases after middle age, the Global Burden of Disease (GBD) Study 2021 estimated that age‐standardized CVD death rates are between 250 and 400 deaths/100,000 people for most LMICs and <150 for HICs and that premature mortality is increasing at younger ages [61]. In China, CVD mortality has steadily increased since the late 1990s. By 2023, over 4 milli on people die from CVD each year—nearly half of all deaths—with coronary heart disease (CHD) and stroke as the main causes [62]. India and Bangladesh face similar crises: 30–35% of people have hypertension, over 60% is uncontrolled, and hospital MI death rates exceed 60%. Sub‐Saharan Africa's MI mortality is even higher than in Europe or North America [63]. The risk factor tsunami begins earlier: approximately 34.3% of Chinese adults are overweight/obese, 12.4% (approximately half undiagnosed) have diabetes, and smoking rates among young men in South Asia are more than 30%, accompanied by the accumulation of ambient air pollution into an extra layer of threat to most inhabitants on Earth [64]. The annual average PM2.5 levels across China and India have repeatedly exceeded 40–60 µg/m^3^, which can be as high as 8–12 times the WHO guideline of 5 µg/m^3^ [65]. It is estimated that PM2.5 exposure leads to approximately 10–15% of total CVD deaths [66]. LMICs face a “double burden” of persistently high acute mortality and insufficiently prepared chronic care systems [67].

By assessing the data in terms of DALYs, there was more dramatic separation in the two‐decade time period being compared: between 1990 and 2019, for example, HICs dropped CVD‐attributable DALYs by approximately 30%, mostly by forestalling premature deaths, but years lived with disability for HF and AF spiked—an approximately 40% increase in US HF‐related DALYs between 2000 and 2020 [68]. Moreover, total CVD DALYs in LMICs continued to rise; Southeast Asia, South Asia, and sub‐Saharan Africa experienced the greatest increase in numbers between 1990 and 2019 (55%) [1, 69, 70, 71]. In that region, acute conditions (MI, stroke) were responsible for an astounding 74% of DALYs in South Asia and contributed more than 34% of new cases since 1990 [72]. In addition, sub‐Saharan Africa has >39 million rheumatic heart cases, an average age of onset of 50 years, and a 2‐year mortality >50%, leading to high levels of DALYs annually [70].

The globe today is split into two categories: “slower mortality decline+rising chronic disability” in rich countries, contrasted with “continued mortality increase+acute fatality dominance” in LMICs. Future CVD approaches will thus have to be differentiated and targeted: HICs should be targeted toward chronic disease prevention and rehabilitation while LMICs should be targeted to improve their emergency services, nip risk factors in the bud, and prevent premature disease onset.

### 2.3 CVD Spectrum

Driven by the growth of global socioeconomic development, population aging, and care innovations, the CVD spectrum is shifting from acute, fatal events such as ST‐elevation MI (STEMI) and hemorrhagic stroke to chronic, nonfatal events such as HF, chronic coronary syndrome (CCS) and AF [73]. Among HICs, hospitalized cases of AMI fell dramatically from >20% in the early 1970s to <5–7%, according to the AHA (2023) [74]. Nevertheless, the prevalence of HF > 64 M currently exists, with HF‐related DALYs increasing almost 40% in 2 decades [75]. In LMICs, acute events, such as those in China, accounted for ∼46% of the 2019 deaths attributable to CVD, more than 80% of which occurred because of stroke and coronary disease [76].

The molecular underpinning is the cumulative lifetime exposure to risk factors [77]. Episodic hypertension activates the RAAS and NADPH oxidase, which results in endothelial dysfunction and small‐artery remodeling [78]. Chronic hyperglycemia can cause AGE–RAGE‐dependent activation of CMKCT kinase, promoting myocardial fibrosis [79]. Moreover, it can lead to transformation from acute to chronic features through AMPK/FOXO3a signal transduction via beta cells [80]. CHD involves these steps [81]. In Europe and North America, the rate of 30‐day mortality after STEMI in clinical practice is currently less than 5%, but postischemic HF rates are much higher, affecting approximately 8–10% of the population over the age of 65 years [82]. Survivors have persistent mitochondrial dysfunction and abnormal calcium handling, providing conditions for maladaptive cardiac remodeling [83].

Strokes in East Asia remain disproportionately severe, with China's age‐standardized incidence rate at 246 per 100,000, which is twice the rate of Western populations (∼120 per 100,000), and hemorrhagic strokes represent more than 30% of the cases compared with less than 15% in Europe/North America [84]. Long‐term AT1‐receptor signaling in hypertensive vascular smooth muscle through hyaline arteriolosclerosis results in cerebral aneurysms and explains the excess hemorrhagic load [85]. AF now stands out as the defining arrhythmia of aging communities, with its prevalence surpassing 15% among Americans older than 80 years, and it quintuples the risk of stroke [86]. The remodeling of atrial fibrosis and its electrophysiology involving TGF‐β/Smad‐mediated collagen deposition and reduced expression of Nav1.5/Kv1.5 provide the stage for a thromboembolic prone environment [87].

The causes of HF differ greatly among different areas [88]. In HICs, ischemic cardiomyopathy and hypertension‐induced remodeling are more common, whereas rheumatic valvular disease accounts for 30–40% of all cases of HF in sub‐Saharan Africa and South Asia, with a median age of onset of <50 years and a 2‐year mortality of over 50% [89]. Hypertension accounts for approximately 60% of new‐onset HF cases in China, whereas coronary disease accounts for 70% of new‐onset HF cases in Western cohorts [90]. Studies by molecular phenotyping have shown that HFpEF involves microvascular inflammation and NO deficiency and that impaired cardiomyocyte energetics and deficient mitophagy are hallmarks of HFrEF [91]. Therapeutic gaps exist: only ∼28% of Chinese AF patients are given oral anticoagulants, whereas ∼60–70% of European or North American patients perpetuate stroke [92]. Consequently, HICs must target slowing chronic progression and preserving quality of life, whereas LMICs need aggressive early control of hypertension, diabetes, and rheumatic heart disease. Mechanistic insights point to future precision strategies centered on endothelial protection, antifibrotic therapy, and metabolic reprogramming of the myocardium. The current epidemiological status of CVD is summarized in Table 1.

**TABLE 1:** Current epidemiological status of CVDs.

Dimension	High‐income countries	Middle‐income countries	Low‐income countries	References
Overall burden pattern	Declining CVD mortality with rising chronic disability	Emerging global epicenter of CVD burden	Predominantly acute, highly fatal CVD events	[14, 15, 16, 17, 18, 19, 20]
Age‐standardized CVD mortality	<150 per 100,000; 40–60% decline since 1970s (United States −53%, United Kingdom −46%)	Rising since the 1990s; >4 million deaths/year in China (∼43% of total deaths)	350–400 per 100,000; substantially higher than HICs	[24, 33, 39, 40]
Dominant disease types	Chronic HF, chronic coronary syndrome (CCS), atrial fibrillation (AF)	Acute stroke and CHD plus rapidly expanding chronic disease pool	Acute MI, stroke, rheumatic heart disease	[18, 23, 32, 76]
In‐hospital AMI mortality	5–7%	Urban 6–8%; rural >15%	>20%, 3–4 times higher than HICs	[27, 34, 46]
Disease spectrum shift	From fatal acute events to chronic nonfatal conditions	Coexistence of acute fatality and chronic disease expansion	Still dominated by early‐onset, high‐fatality acute disease	[52, 73]
Heart failure (HF) burden	>6.5 million patients (projected 9 million by 2030); HF‐related DALYs ↑ ∼40%	Hypertension causes ∼60% of new HF cases	Rheumatic disease accounts for 30–40% of HF; onset <50 years	[28, 59, 89, 90]
Atrial fibrillation (AF)	>9% prevalence in ≥65 years; >15% in ≥80 years	Oral anticoagulant use ∼28%	Data limited; stroke risk remains high	[29, 86, 92]
Stroke burden	Mainly ischemic stroke	Stroke accounts for ∼30% of DALYs in 40–60 years age group	Stroke mortality 2–3× higher than in HICs	[37, 47, 50]
Hemorrhagic stroke proportion	<15%	>30%; incidence ∼246 per 100,000	Relatively high	[84, 85]
Major risk factors	Obesity (>40%), diabetes (∼11%), population aging	Hypertension (27.5%, control 15%), smoking, diabetes (>12%), air pollution	Poor hypertension control (<20%), infection‐related heart disease, poverty	[16, 37, 42, 43]
Environmental factors	Relatively lower pollution exposure	PM2.5 often 40–60 µg/m^3^ (8–12× WHO guideline)	Significant exposure plus weak infrastructure	[41, 64, 65, 66]
DALYs trend	Total CVD DALYs ↓ ∼30% (1990–2019), but YLDs ↑	Total DALYs continue to rise	Rapid DALY growth dominated by acute events	[68, 69, 72]
Population disparities	Mainly older adults	Marked urban–rural, sex, and regional heterogeneity	High burden in younger populations	[45, 48, 49, 70]
Public health challenge	Shift from “preventing death” to “preventing disability”	Dual pressure: acute care + long‐term management	Urgent need to strengthen emergency care and prevention	[31, 38, 44]
Priority strategies	Chronic disease management, rehabilitation, QoL preservation	Risk‐factor control + acute care + lifelong management	Hypertension control, rheumatic disease prevention, stroke/MI emergency care	[51, 60, 67]

*Abbreviations*: CVD, cardiovascular disease; HF, heart failure; CCS, chronic coronary syndrome; AF, atrial fibrillation; CHD, coronary heart disease; AMI, acute myocardial infarction; DALY, disability‐adjusted life year; YLD, years lived with disability; PM2.5, particulate matter ≤ 2.5 µm; QoL, quality of life.

## 3 Classification and Mechanism of Risk Factors

**FIGURE 2:**
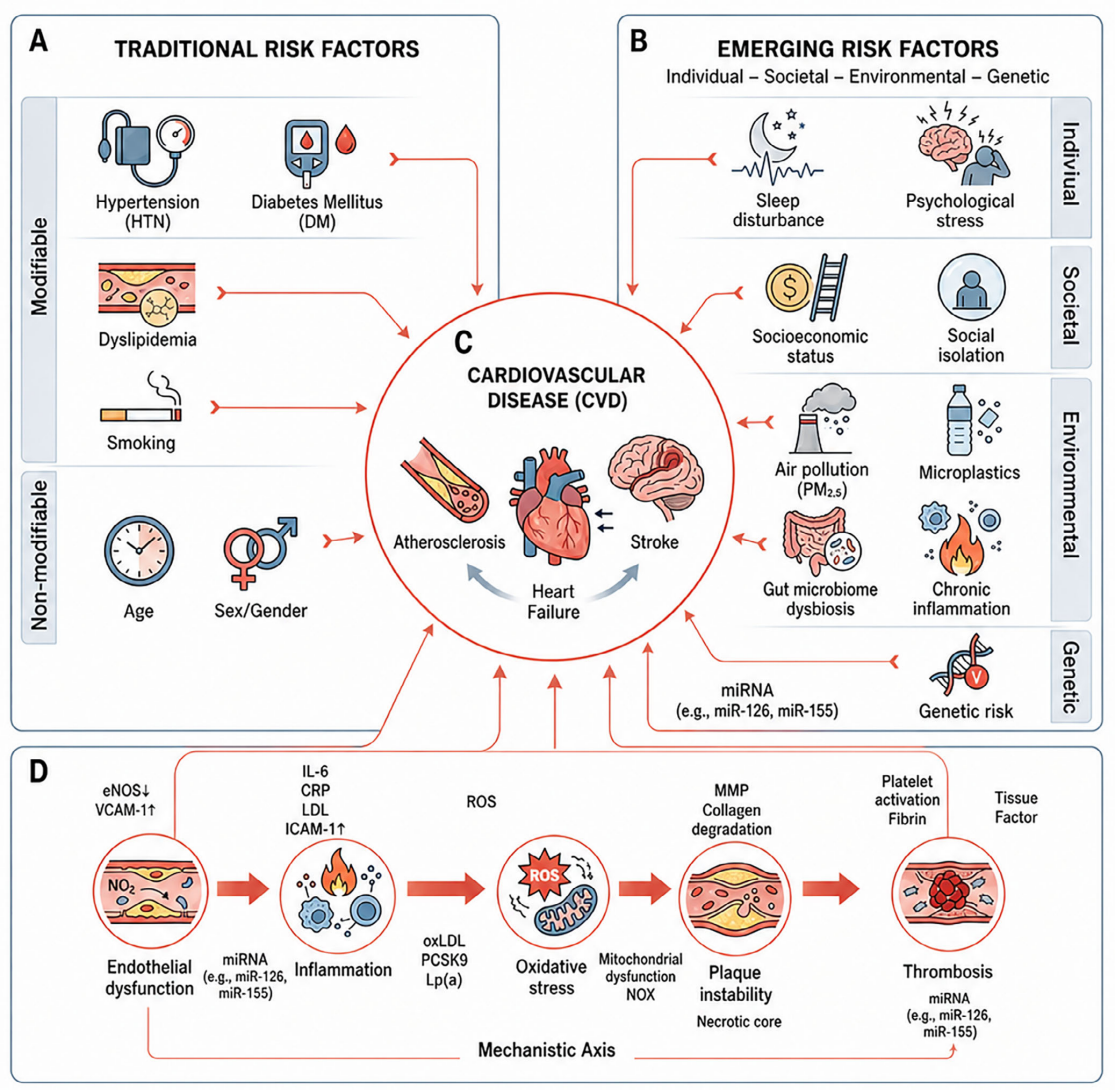
Systems‐level landscape of cardiovascular risk factors: integration of traditional and emerging determinants through molecular nodes and a mechanistic axis driving CVD progression. (Draft diagram created in WPS office PPT with GPT 5.5‐assisted layout suggestions. Final image refined and annotated by the authors. All pathway details were cross‐checked against experimental data.) (A) Hypertension, diabetes, dyslipidemia, smoking, obesity, age, sex, and physical inactivity. (B) Sleep disorders, stress, socioeconomic factors, PM2.5, microplastics, gut dysbiosis, chronic inflammation, and genetic susceptibility. (C) Endothelial dysfunction, inflammation, oxidative stress, mitochondrial dysfunction, plaque instability, and thrombosis. (D) From risk exposure to vascular injury, atherosclerosis, myocardial infarction, stroke, and heart failure.

The evidence suggests that the CVD burden continues to grow and that its risk determinants are distributed unevenly among different regions of the world. Both the World Health Organization (WHO) and the GBD group indicate that the largest modifiable driver of CVD is increased blood pressure. Over one billion adults are hypertensive, yet only 20% receive adequate treatment for their condition [93]. The prevalence of hypertension remains high in low‐ and middle‐income settings, while obesity and T2DM are spreading rapidly as part of the inevitable process of urbanization and “Westernization” of diets, accompanied by a shift to obesity due to physical inactivity [94]. Large Chinese cohorts (e.g., China Kadoorie Biobank [CKB], China‐PAR and 4C) show that hypertension, smoking, high‐sodium intake, physical inactivity, and adiposity are the main drivers of CVD risk in China—which cumulatively increases risk—with an almost exponential growth in the hazard function when multiple factors are present [95]. CVD arises from the cumulative impact of traditional and emerging risk factors across individual, societal, environmental, and genetic domains. Collectively, these determinants converge on shared pathophysiological pathways, linking exposure to endothelial dysfunction, inflammation, oxidative stress, plaque instability, and thrombosis, ultimately leading to major clinical phenotypes, including atherosclerosis, stroke, and HF (Figure 2).

The GBD 2019 study revealed that 68% of all causes of death can be attributed to 20 modifiable risk categories [96]. Hypertension, smoking, ambient air pollution, fasting plasma glucose level, and BMI account for the largest shares of total burdens across the spectrum of risk factors. Although rigorous control of these traditional determinants can avert over 40% of CVD deaths, an important residual risk still remains [97]. Hence, the quest for precision medicines has advanced.

Genetic epidemiology points to inflammatory biomarkers, prothrombotic predispositions, vascular biological attributes, and gene–environmental interactions contributing substantially to the unexplained part of the variation not attributed to classic factors [98]. Population‐level social determinants (e.g., economic disparities, educational divides, and heterogeneity in the pace of urbanization) may drive geographical variations in CVD risk.

### 3.1 Traditional Risk Factors

In the last decade, cardiovascular studies have shifted their focus from single risk factor control to integrated multipathway and multitarget prevention and treatment. Atherosclerosis is not only caused by elevated blood pressure per se but also promoted by the phenotypic switching of vascular smooth muscle cells (VSMCs), leading to increased stiffness and arterial remodeling with loss of function of the vessel's tunica media [99]. Genome‐wide analysis revealed that variants located at ATP2B1, UMOD, SH2B3, and so on, play critical roles in BP regulation, and even small genetically increased pressure levels greatly increase the event rates of CVDs among middle‐aged people and older individuals [100, 101]. Therefore, the future trend of management should focus on vascular protection rather than lowering blood pressure [102].

Pathophysiology redid the classification of risk: Lp(a) is now recognized as an independent often familial determinant, which confers a twofold increase in risk within affected individuals, whereas triglyceride‐rich remnants (VLDLs, IDLs) link insulin resistance with inflammation and account for much of the residual risk after broad‐spectrum lipid‐lowering agents are used [103, 104]. Both CANTOS and COLCOT trial data showed that targeted anti‐inflammatory therapy reduced CV events, thus providing enough evidence for the modulation of inflammation in the CV [105]. Hyperglycemic fluctuations and recurrent hypoglycemia increase the likelihood of developing vascular complications; therefore, having smooth CV glucose excursion should be more beneficial than lowering HbA1c alone [106]. GLP‐1RAs and SGLT2Is protect the CV system, which also favors this conclusion [107].

Lifestyle factors have been reconsidered at the mechanistic level; smoking accelerates endothelial apoptosis through oxidative stress and miRNA dysregulation, and e‐cigarettes are not safer than tobacco products [108]. The evidence refutes the J‐shaped alcohol curve, especially for ALDH2 loss‐of‐function carriers [109]. Emerging studies have shown that visceral adiposity releases proinflammatory cytokines, maintaining a state of chronic low‐grade inflammation in which sedentary behavior is recognized as a considerable hazard, separate from the adverse effects of a high‐salt diet, shifting the gut microbiota toward Th17 activity, versus increased dietary fiber intake, which enhances barrier function and promotes SCFAs (short‐chain fatty acids) with vascular benefits [110]. Collectively, these findings underscore that cardiovascular risk is driven by interconnected metabolic, inflammatory, and lifestyle pathways, highlighting the necessity of integrated and mechanism‐based preventive strategies.

### 3.2 Emerging Risk Factors

#### 3.2.1 Environmental Factors: Air Pollution, Noise, and Climate Change

In the last decade, cardiovascular studies have shifted their focus from single risk factor control to integrated multipathway and multitarget prevention and treatment. Atherosclerosis is not only caused by elevated blood pressure per se but also promoted by the phenotypic switching of VSMCs, leading to increased stiffness and arterial remodeling with loss of function of the vessel's tunica media [111]. Genome‐wide analysis revealed that variants were located at the loci of ATP2B1, UMOD, SH2B3, and so on [112] and play a critical role in BP regulation, and even small genetically increased pressure levels greatly increase the event rates of CVDs among middle‐aged people and older individuals [113]. Therefore, the future trend of management should focus on vascular protection rather than lowering blood pressure.

Noise exposure is also closely related to the occurrence of ischemic heart disease and hypertension; even traffic noise below set limit values increases the risk of these diseases. Short‐term interventions have shown that short exposure (several hours) to nighttime aircraft noise impairs endothelial functions and sleep quality [114]. Furthermore, in animal models, vascular inflammation is also reduced upon withdrawal from noise exposure. Several weeks may be necessary for high BP to return to baseline levels after exposure ceases. In occupational cohorts, chronic exposure of service and transport workers to noise and aerosols has significantly increased hypertension risk [115]. Mechanistically, noise and air pollutants can increase BP and promote arterial stiffening and autonomic imbalance by enhancing sympathetic–HPA axis activation, circadian desynchronization, and oxidative stress pathways in a synergistic manner with air pollution [116].

Extreme heat caused by climate change is now acting as a “new normal” with respect to CVD burden. In Europe, there are over 48,000 heat‐related deaths, and US surveillance similarly identified a sharp increase in emergency department visits during heat waves, where females, elderly individuals, and people with preexisting CVD are at greater risk [117]. Furthermore, heat waves often coincide with exposures to compound pollutants, such as wildfire smoke and ozone pollution, which in turn increase the risk and put pressure on healthcare system infrastructure.

#### 3.2.2 Social and Psychological Factors: Chronic Stress, Depression, and Social Isolation

Social determinants such as chronic stress, depression, and social isolation are not simply comorbidities or risk factors for CVD; they are actually the drivers of CVD onset and progression. HF patients exhibit much higher mortality after suffering psychological burdens from natural disasters: postearthquake depression was associated with substantially higher mortality among earthquake survivors with HF, according to one report in 2023 [118]. These effects include high levels of upregulated inflammatory cytokines (IL‐6, CRP), autonomic imbalance, and reduced nocturnal heart rate variability [119]. Socioeconomic status matters as well: a one percentage point increase in unemployment causes an approximately 6% increase in hospital admissions for HF, meaning that socioeconomic shocks are modifiable CVD triggers [120]. Chronic social isolation (being alone)—studied via longitudinal cohorts and natural experiments—is another known trigger of CVD [121]. Having no intimate ties or community support leads to a heightened chance of facing heartbeat issues such as HF, MI, and stroke, especially with preexisting CVDs [122]. Mechanisms may include disrupted sleep, harmful lifestyles, and continuous activation of the HPA axis and sympathoadrenal systems, which can lead to hypertension and arrhythmia [123]. Depression is thought to be a “toxic” comorbidity; systematic reviews and meta‐analyses have shown that there is a dose‒response relationship between depression and CVDs [124]. On the basis of its magnitude of harm to patients, IP is correlated with a higher PA–CS mortality/readmission rate and more severe manifestations of endothelial dysfunction and inflammation [125]. Specifically, it underlines public health pathways and clinical pathways that should (1) screen for mental health and social support needs as part of primary and secondary CVD prevention efforts, especially in circumstances of unemployment growth or unrest; (2) evaluate the long‐term benefits of cognitive behavioral therapy, mindfulness training, and social interventions for cardiac health outcomes and prognosis; and (3) enhance unemployment insurance, housing stability, and community service provisions at the policy level while incorporating mental health support provisions into disaster response procedures, with the aim of reducing psychosocially induced CVD burdens [126]. Taken together, these findings highlight psychosocial and socioeconomic factors as integral and modifiable drivers of CVD, underscoring the need for their systematic integration into both clinical care and public health strategies.

#### 3.2.3 Inflammation and Infection: Chronic Inflammation, Periodontitis, and Viral Infections

We no longer consider a marginal idea; now, we know that inflammation and infections play key roles in CVD's cause and how they develop. The past 3 years have seen a vast body of new research being conducted, with more evidence of the different ways in which inflammation and infections affect the course of events and initiate action for treatment.

Chronic low‐grade inflammation results from metabolic disorders or certain environmental or lifestyle elements, such as obesity, insulin resistance, cigarette smoking, and air pollution, which lead to endothelial dysfunction, early formation of atherosclerotic plaques and increased plaque vulnerability [127]. A multinational cohort study published in 2024 demonstrated that both high‐sensitivity CRP (hs‐CRP) and interleukin‐6 (IL‐6) levels were still high in people who did not have any typical risk factors; even so, these levels were strongly associated with future cardiovascular incidents, and the degree of plaque inflammatory signal or atherosclerosis burden in coronary arteries determined the strength of the association between hs‐CRP and IL‐6 levels and future cardiovascular incidents [128]. In other words, this research confirmed the predictive value of biomarker levels for patients’ risk assessment in CVD prevention and highlighted the potential use of such biomarkers as targets for imaging or therapy [129].

The effects of chronic oral infection are receiving increasing attention, with more research and concrete evidence in this area [130]. Between 2022 and 2024, a number of meta‐analyses and case‒control works indicate that moderate to severe periodontitis results in a much greater risk for MI, stroke, and peripheral artery disease [131]. Treating periodontitis (using mechanical debridement combined with targeted antibiotics) has been proven to reduce gingival crevicular IL‐1β, tumor necrosis factor‐α (TNF‐α), and IL‐6 levels by up to 6–12 months, together with an improvement in FMD [132]. Only a single randomized clinical trial reported that, after 6 months of intensive dental care, periodontal treatment lowered oxidized low‐density lipoprotein (LDL) and PWV in subjects with stable coronary disease [133]. Thus, the maintenance of good oral health may improve systemic vascular health [134].

Since the onset of the SARS‐CoV‐2 pandemic, viral infection has gradually drawn everyone's attention [135]. Apart from acute myocarditis, endotheliitis, the so‐called “long‐COVID” or “long‐COVID” stage carries a risk lasting 1 year: the risk of permanent arrhythmia, HF, and left ventricular dysfunction that remains high; these risks are high because there continues to be low‐grade inflammation, microvascular impairment, and T‐cell dysregulation [136]. Additionally, postinfectious cardiovascular events can be triggered in older persons and in individuals with preexisting CVD not only by influenza and other respiratory viruses (such as RSV and postviral *Streptococcus pneumoniae* superinfections) but also by SARS‐CoV‐2 infection and variants [137]. Increased vaccination coverage, early postinfectious management, and judicious use of antivirals can substantially reduce these cardiac complications [138].

#### 3.2.4 Genetics and Epigenetics: Family History, GWAS Findings, and Noncoding RNA

Over many years, several genetic or epigenetic factors concerning CVD advancement and progression have been focused on and are under intense study; advances made over the last 3 years have helped us expand our knowledge of various CVD aspects, such as family history, genome‐wide association studies (GWASs), and noncoding RNAs [139]. Family history has long served as a proxy for genetic factors; however, it cannot provide insights into causative mechanisms yet still plays a very important role in identifying disease risk levels [140]. New findings have revealed that single nucleotide polymorphisms (SNPs) at the 9p21 locus are major players in population‐mediated coronary artery disease (CAD) predisposition, highlighting that family history is not an indication of the presence of one mutation but rather a synthesis of multiple gene variants interacting within a given environment [141].

In the last decade, GWASs have revealed hundreds of loci associated with CVD, whereas more recent large‐scale transethnic studies have revealed some population‐specific risk genes [71]. In Asian GWASs, some loci not previously described in European cohorts have been identified [142]. These include novel gene regions related to blood pressure regulation and lipid metabolism [143]. These discoveries have helped populate the world map of CVD genetics with greater genomic detail, enabling more individualized risk assessment and informed risk management strategies [144]. Moreover, knowledge about functional effects at the gene level has led to the identification of PCSK9 and LPA variants that can be used in targeted drug development and even in the clinic, indicating great promise for the potential future use of genetics for precision medicine [145].

Moreover, with the emergence of epigenetics, people now understand how complicated the role of noncoding RNAs is and how they could be considered much more broadly [146]. Among them, microRNAs such as miR‐21, miR‐92a, and miR‐155 are well‐known candidates for determining the course of atherosclerosis, HF, and the inflammatory response [147]. In a clinical trial, it was shown that miR‐21 stimulates smooth muscle cell proliferation and accelerates plaque formation by modulating TGF‐β signaling, whereas circulating miR‐92a favors cardiovascular remodeling and induces adverse cardiovascular events through destabilization of the endothelium [148]. As our testing methods improve, miRNAs circulating in blood are currently under investigation as powerful potential biomarkers that may help us diagnose CVD at an earlier stage or allow us to stratify CVD patients properly [149].

Long noncoding RNAs (lncRNAs) are currently an important topic of interest [150]. Both MALAT1 and H19 are potential lncRNAs that play a role in the regulation of gene expression in cardiac hypertrophy and HF through crosstalk with endogenous RNA [151]. H19 is considered a major player in genetic susceptibility, as it is related to the 9p21 risk locus [152]. It has also been proposed that some lncRNAs, such as ANRIL, are likely to have promising functions as potential therapeutic targets for controlling cell cycle progression and/or regulating the inflammatory signal transduction pathway [153].

#### 3.2.5 Others: Gut Microbiota, Nanoparticle/Toxin Exposure

Studies exploring the associations between the gut microbiota or nanoparticle/toxin exposure and CVD have revealed several new pathogenic processes that might lead to potential therapeutic approaches involving targeting the gut microbiota [154]. In turn, the gut microbiota is reported to be involved in CVD risk via different routes, such as altering the abundance of specific microbially derived metabolites such as TMA and its oxidation products (nontoxigenic *Toxoplasma gondii*), which contribute to the activation of the toll‐like receptor 4 pathway [155]. Trimethylamine N‐oxide (TMAO) is the oxidation product of TMA, promotes the deposition of cholesterol, and enhances platelet aggregation and inflammation to accelerate atherosclerosis; conversely, SCFAs formed by beneficial bacteria improve the function of endothelial cells, reduce oxidative stress, and regulate blood pressure and glucose metabolism, which has a protective effect on the human body [156]. Clinical cohort studies in 2022 and 2023 revealed that gut microbe diversity is negatively correlated with CAD severity and that the composition of gut microbes affects the prognosis of HF patients and may indicate susceptibility to disease [157]. All these studies have provided the underlying theoretical foundation for the exploration and application of probiotics, dietary intervention, and fecal microbiota transplantation in CVD prevention and treatment [158].

Currently, nanoparticle exposure and toxins represent new cardiac risk factors [159]. NPs from air pollutants such as PM2.5 induce plaque development by stimulating inflammation in the endothelium and generating oxidative stress; these particles simultaneously damage blood vessels through a decrease in stability, which affects VSMCs via migration or proliferation [160]. Prognostic studies published in 2022–2023 revealed that they significantly increase the burden on the heart for high‐risk patients [161]. Long‐term exposure to environmental pollutants (such as lead, cadmium, pesticides, and plasticizers) can significantly increase the incidence of cardiovascular events, which is likely related to promoting oxidative stress, altering lipid metabolism and inducing epigenetic changes, including DNA methylation and histone modification [162]. Emerging studies have shown that nanoparticle exposure can affect the epigenetic state of cardiovascular‐related genes, with long‐term adverse impacts on vascular function and cardiac structure [163]. This presents both a risk and an opportunity for nanoparticle‐based precision drug delivery systems and targeted therapy for CVD [164].

### 3.3 The Aggregation and Interaction of Risk Factors

Metabolic syndrome (MS) is not a mere clustering of obesity, hyperglycemia, hypertension, and dyslipidemia; instead, it is a disease characterized by an integrated action of insulin resistance, chronic low‐grade inflammation, oxidative stress, and endothelial dysfunction [165]. Recent studies from 2022 to 2023 have shown that the plasma IL‐6 and TNF‐α levels of MS patients are increased by 1.7–2.4 times compared with those of normal control subjects, and for each 1 pg/mL increase in the serum IL‐6 concentration, endothelium‐dependent vasodilation decreases by 0.9% (*p* < 0.01), suggesting that the “inflammation–vascular axis” is a key pathway [166]. Dysfunction of adipose tissue plays a critical role in the two abovementioned diseases [167]. In obese subjects with MS, macrophage infiltration into adipose tissue may increase up to 40% (≤10% in normal individuals), leading to a 3.5‐fold increase in the ratio of leptin/adiponectin, which increases the PWV by 1.2 m/s, indicating accelerated arterial stiffening [168]. Longitudinal cohort studies revealed that in the following 5 years, patients with MS developed a left ventricular hypertrophy incidence that was 1.8 times greater and an incident diastolic dysfunction occurrence risk that was 2.3 times greater than that of controls as well as a faster 0.8 mL/min/1.73 m^2^ per year eGFR annual decline—indicating independent heart and kidney target organs for MS [169]. NAFLD is observed in up to 58% of people with MS versus 18% of those without MS, and with a 13% increase in liver fat content, cardiovascular event risk increases by an additional 13% [170]. These changes establish a “liver–heart–kidney” organ injury network associated with MS pathogenesis [171].

In this network, multiple risk factors have a synergistic effect and lead to the amplification of cardiovascular risk in a nonlinear way [172]. A 2019 meta‐analysis of 380,000 people revealed that hyperglycemia (HbA1c ≥ 6%), hypertension (≥130/85 mmHg), and obesity (BMI ≥ 30 kg/m^2^) all work together and cause the incidence of CHD to be 2.7 times as high as that of the individual (95% CI, 2.3–3.1) rather than the mathematically additive 1.9 times the chance, indicating that hyperglycemia increases the pathogenic effect of all factors by 0.8‐fold [173]. Mechanistically, hyperglycemia increases endothelial ROS production by 60%; when combined with hypertension, Collagen IV is deposited 2.4‐fold, accelerating arteriosclerosis; hypercholesterolemia (LDL‐C ≥ 3.4 mmol/L) with obesity enlarges the vascular lipid core area 1.6‐fold and increases the plaque vulnerability index by 40% [174]. A posthoc analysis of 22,000 patient randomized controlled trials (RCTs) published in 2021 also revealed that intensive glucose lowering or BP control alone decreased cardiovascular endpoints by only 6 and 8%, respectively, whereas a comprehensive intervention (glucose lowering + BP control + lipid reduction + weight loss) decreased cardiovascular risk by 24% (*p* < 0.001), indicating that “single‐target” strategies fall short and that multiple risk factor modifications are needed [175].

These combined effects are strengthened by environmental factors [176]. The results from multicenter research in the past 2 years indicate strong additive associations among air pollution and traditional risk factors, in which, for every yearly average PM2.5 concentration of ≥35 µg/m^3^, hypertensive patients present with an extra 1.4 mmHg increase in SBPV and a 1.5‐fold greater risk of stroke, and the risk is increased by 2.8 times when stroke suffers from MS comorbidities owing to PM2.5 exposure, which reduces vascular eNOS activity by 30%, together with inbuilt endothelial dysfunction, increases the reduction in arterial compliance by 8%, whereas the progression of annual intima‒media thickness (IMT) thickening in MS patients is 0.02 mm per 10 ppb NOx greater than that in non‐MS patients, doubling their rate [177]. Elderly MS patients (aged ≥ 65 years) are particularly prone to this disease [178]. For each 10 µg/m^3^ increase in PM2.5, the risk of hospitalization for AMI for MS is greater (3.2%) than that for non‐MS individuals (1.7%), indicating that given the absence of a cardioprotective effect from NSAIDs or the A2A AR antagonist CGS21680, future CVD prevention and management strategies might integrate “metabolic–environmental” (metabolic and environmental) approaches, such as increasing monitoring of blood pressure and inflammation among MS patients under high‐pollution conditions and prioritizing air purification and emission reductions to interrupt the loop amplifying the vicious cycle between the environment and metabolism [179].

## 4 Screening and Risk Assessment

**FIGURE 3:**
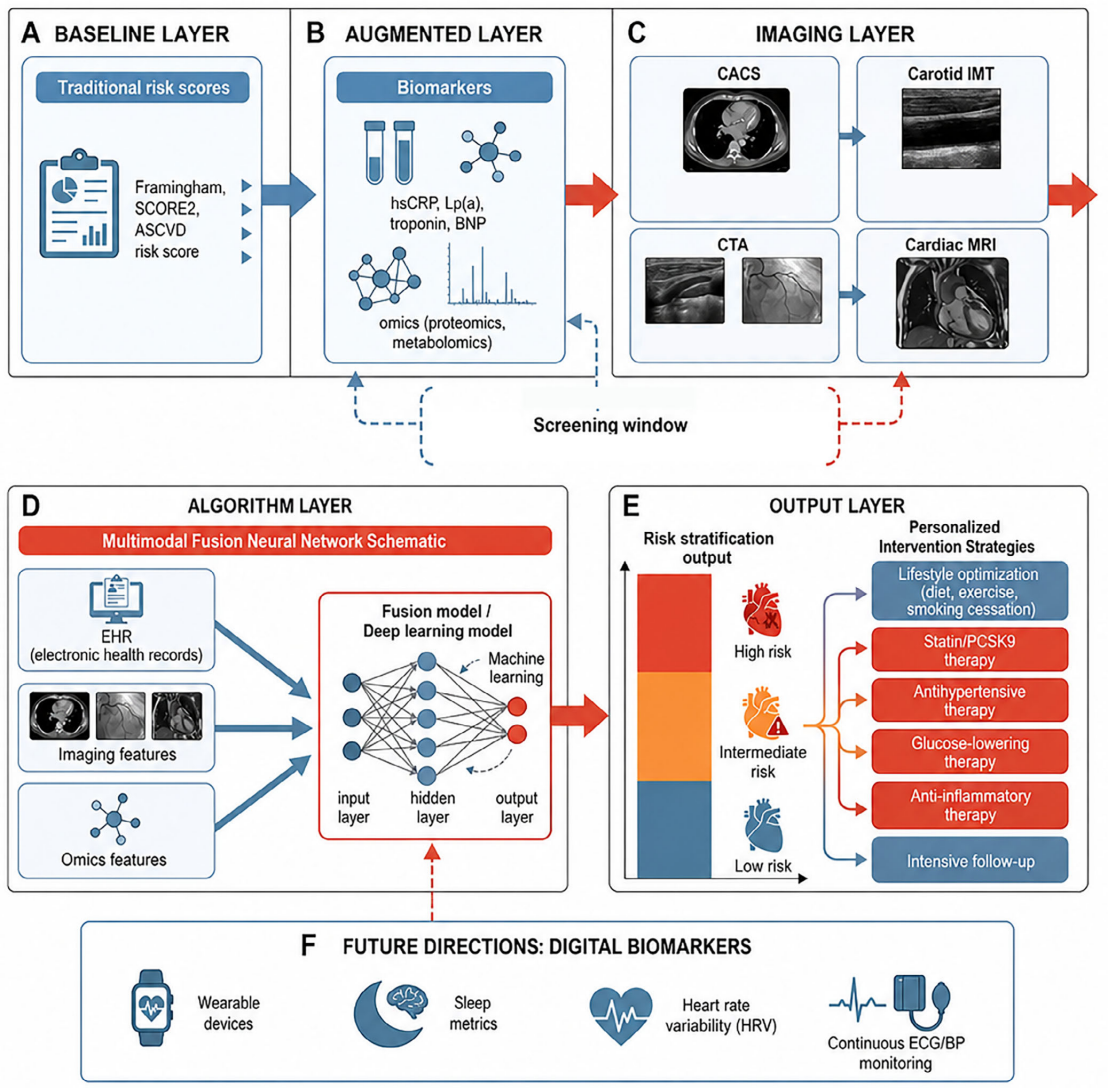
Clinical pipeline for CVD screening and risk assessment: from traditional risk scores to AI‐driven multimodal prediction and personalized intervention. (Draft diagram created in WPS office PPT with GPT 5.5‐assisted layout suggestions. Final image refined and annotated by the authors. All pathway details were cross‐checked against experimental data.) (A) Framingham, SCORE2, and ASCVD used to estimate baseline cardiovascular risk. (B) hsCRP, Lp(a), troponin, BNP, and multiomics panels for enhanced risk detection. (C) Coronary artery calcium score (CACS) and coronary CTA to assess structural disease. (D) Carotid intima‐media thickness (IMT) and cardiac MRI for functional and anatomical evaluation. (E) Combining multimodal data to optimize risk stratification and guide personalized prevention. (F) Wearable devices, heart rate variability (HRV), and continuous monitoring to enable dynamic risk assessment and early intervention.

Screening and risk assessment represent the critical interface between the epidemiological burden and precision prevention. Contemporary clinical evaluation is evolving from conventional risk scoring to a multimodal approach that integrates biomarkers, advanced imaging, and artificial intelligence (AI)‐enabled data fusion. As summarized in Figure 3, this stepwise pipeline enables refined risk stratification and supports individualized intervention strategies while defining a screening window for intensified testing and highlighting future integration of digital biomarkers.

### 4.1 Basic Screening and Population Strategies

Cardiovascular risk screening has entered an era of “four‐dimensional dynamic + multiomics” composite phenotypes as opposed to the classical “low‐cost–high‐resolution” model [180]. The META‐4D study (European Society of Cardiology [ESC], 2023; *n* = 1.2 M) revealed a considerable increase in the net reclassification improvement in the prediction of CHD in persons <40 years old for those who had a 6‐month at‐home SBP‐CV over baseline, in addition to conventional biosignals and self‐reports [181]. It is speculated that blood pressure stability might be more sensitive for detecting early vascular aging than absolute blood pressure levels are [182]. With respect to a Mendelian randomization analysis from the UK Biobank (*n* = 43,000), the causality ascribed to LDL cholesterol (LDL‐C) exposure to the development of coronary artery calcium (CAC) score decreased according to age. Among subjects younger than 40 years, each 0.5 mmol/L reduction in LDL‐C during their lifetime was associated with a decreased 28% lifetime risk for MI, but this association disappeared in those above 60 years of age, where merely a modest decrease of 7% was observed [183]. In accordance with these findings, the ESC guideline 2025 recommends the inclusion of individuals aged 18–39 years who have LDL‐C levels ≥3.0 mmol/L and in the highest 20% of PRSs among primary prevention candidates [184].

The avi‐DPP study (China, *n* = 28,000) revealed that screening for prediabetes with both HbA1c and OGTT results in the identification of 31% more prediabetes cases than does the OGTT alone, and cardiovascular events are reduced by 9% after 5 years [185]. Screening with HbA1c plus OGTT followed by lipid subfraction > AIP 0.06 allowed the correct stratification of high‐risk patients, defined as those with coronary flow reserve <2.5, who needed intervention to normalize coronary perfusion or stenting/PCI [186]. Thus, the Chinese Expert Consensus on Diabetes Prevention recommends that individuals ≥30 years old should undergo combined “HbA1c + lipid subfraction” testing every 2 years, with an ICER of $21,400/QALY [187].

The idea of BMI‐years (i.e., accumulating overweight time during childhood, adolescence, adulthood, and middle age) was shown to be valid via the ARIC data from 2024, demonstrating that for every 1‐SD increase in cumulative exposure, the LVMI increased by 4.2 g/m^2^, and the risk of AF was 22% greater, leading to its integration into the AHA's 2025 “Life's Essential 8” risk score model [188]. In contrast, the total fat mass percentage (TFM%, based on 3D scanning) demonstrated better predictive ability than BMI among Asian populations [189]. A Korean cohort study from 2024 reported that every 1% increase in body fat was associated with a 6% increased risk of MI among men and a 9% increased risk among women, indicating the potential importance of lean body mass [190]. Smartphone‐based AI body fatness measurements can be used for low‐cost and wide‐scale applications [191].

The HEALTH‐RI (2025) program advocated the adoption of a “precision interval” approach: In those with a PRS < 25th percentile and a 5‐year cardiovascular risk <2.5%, screening intervals can be extended to 5–7 years, whereas in those with PRS > 80th percentile and/or a history of premature familial CVD, rescreening every 1–2 years with coronary CTCa is recommended [192]. This approach reduces the testing burden by 34% and saves approximately €180 million annually in healthcare costs [193]. A grassroots trial across 120 counties in China further validated the feasibility of a “screen–prevent–manage” closed‐loop model: integrating “AI voice follow‐up + home blood pressure cloud platforms” into public health services improved blood pressure control rates among 35–75‐year‐olds from 42 to 61%, yielding a return of ¥6.7 in hospitalization cost savings for every ¥1 invested [194].

### 4.2 Risk Prediction Model

In recent years, machine learning (ML) has begun to be widely used in the prediction of ASCVD risk to overcome the limitations of pooled cohort equations (PCEs) for different ethnicities or people who do not have complete data. For example, a multicohort study utilizing 1.2 million electronic health records from northern California revealed that the gradient boosting method was effective at predicting ASCVD risk in many different populations [195]. The machine gradient boosting machine and extreme gradient boosting models had significantly better C statistics (0.81–0.83) than did the traditional PCE (≈0.79) models across the cross‐validation and independent test sets; in the Asian, Hispanic, and data‐missing subgroups, the classifier performance was significantly better [196]. Crucially, this is the first study to provide evidence that an ML algorithm using nonlinear interactions between variables can decrease the number of people deemed “PCE nonassessable” by 25% and lower the false‐negative rate among high‐risk people, adding a new tool for clinical use [197].

With respect to the external validation and localization of classic models, two independent studies were carried out from 2022 to 2023 and presented new evidence for those with MetS [198]. One such study, which was based in Karachi, Pakistan, reported the following results: the Framingham risk score underestimated 15% of those who were intermediate‐ and high‐risk, and the Globorisk model included diabetes and body mass index, for which the value of the latter index correlated with stroke risk as opposed to CAD [199]. It successfully identified 55.4% high‐risk individuals in South Asians with country‐specific coefficients [200]. In addition, the two models showed a moderate positive correlation (*r* = 0.65), which implies that they may have higher risk stratification accuracy if applied simultaneously in low‐resource settings [201]. Moreover, we conducted a recalibration study on the ASCVD risk of the CKB [202]. After the race coefficient of PCE was corrected to a Chinese‐specific value, the degree of risk overestimation among rural Chinese people or younger Chinese individuals was effectively reduced, and the overall risk estimation was better calibrated [203]. Moreover, in 2021, the 2021 ESC published the SCORE2‐OP model, which is the first to include persons aged 50–89 years from multinational cohorts (*n* = 5.6 million), and the model integrated an age‒BP interaction term for the first time [204]. Compared with previously used models, the new model results revealed a 12% decrease in prediction error for cardiovascular mortality among elderly people; thus, it was incorporated into the latest 2022 ESC Guidelines on Cardiov [205].

Avascular disease prevention targets were evaluated according to whether there were 10‐year overall fatal and nonfatal events in older adults from ten European countries [206]. In addition, in 2023, the updated UK QRISK 3 model added three new predictors, namely, hospitalization with COVID‐19, severe mental illness, and hypertensive disorders during pregnancy, and these changes led to an increase in the C statistic for women of 0.007 points and a decrease in systematic underestimation of 7.3%, which is evidence‐based proof of its advantages in the period after COVID‐19 [207].

### 4.3 Imaging and Advanced Screening

Within the emerging paradigm of “multimodal integration, deep AI embedding, and rapid evidence iteration,” cardiovascular imaging has become a major driver of precision risk stratification. CAC has been established as an independent predictor across multiple cohorts from 2022 to 2024 and has been assigned a new role in “dynamic monitoring” [208]. The MESA‐Advance study demonstrated a dose–response relationship between the CAC progression rate and major adverse cardiovascular events (MACE): individuals with ΔCAC > 100 AU/year had a 2.8‐fold higher event rate [209]. For the first time, the 2024 ESC Guidelines listed CAC progression as a Class IIa recommendation for evaluating intervention effects in intermediate‐ to high‐risk populations [210]. Low‐dose CT combined with DLIR algorithms can reduce radiation exposure to <0.2 mSv with <5% error in Agatston scoring, supporting a “one‐stop” lung cancer screening + CAC assessment approach [211]. Photon‐counting CT (PCCT) enables differentiation between “dense calcium” and “microcalcification” [212]. The PRESTIGE trial revealed that each 1‐log increase in microcalcification burden was associated with a 42% greater long‐term risk of acute coronary syndrome (ACS), indicating that “zero calcium ≠ zero risk” [213].

Routine carotid ultrasound, the 2022 ESH consensus, recommended the use of percentile‐based IMT curves rather than absolute values for age because they reduce the risk of overdiagnosis [214]. Using both superb microvascular imaging (SMI) and shear‐wave elastography (SWE) allows simultaneous morphological‒functional evaluation of cervical atherosclerotic plaques [215]. A carotid ABC score integrating SMI‐neovascularization, SWE‐stiffness, and LDL‐particle count (LDL‐P) significantly improved the prediction of 10‐year MACE [216]. Two‐minute bilateral IMT measurements are possible for primary care with handheld probes using AI‐enhanced automatic contours, pointing to technology decentralization [217]. In the wearable field, AI‐enhanced ECGs and smartwatches permit constant out‐of‐hospital monitoring [218]. The ResNet‐WS algorithm has obtained the highest accuracy result for AF detection, with an F1 score of 0.94 over the MIT‐BIH database, and can identify individuals who are 7.6‐fold more likely to develop AF over a period of 5 years from the Mayo Clinic cohort [NEJM 2023] [219]. The Huawei WATCH D instrument equipped with oscillometric microunch mounted technology fulfilled the ESH guidelines for blood pressure monitoring validated for autonomous use [JACC 2024] while combining the PWTT obtained from PPG with the measurement of the cf‐PWV accomplished through oscillometric microcuff within only 30 s (*r* = 0.89) and a cf‐PWV > 10 m/s 2‐year MACE at a sensitivity of 85% [220].

### 4.4 Biomarkers and Molecular Screening

Owing to cardiovascular imaging and advanced screening, individualized risk stratification is emerging in the paradigm of ″multimodal integration, AI embedding, and rapid evidence iteration. CAC was shown to be an independent predictor from 2022 to 2024 and assumed a vital role in dynamic monitoring in risk assessment [221]. The results revealed that individuals with a change in CAC greater than 100 AU/year had 2.8‐fold greater odds for MACE than individuals with no increase or only a slight increase in CAC [222]. In the assessment of MACE, for the first time, in the 2024 ESC Guideline, intermediate‐ to high‐risk population interventions were recommended for the treatment of coronary artery calcification (CAC) progression (Class IIa) [223]. Low‐dose CT with DLIR (deep learning image reconstruction) can reduce radiation exposure to less than 0.2 mSv and less than 5% scoring error [224]. “One‐stop” lung cancer + CAC screening was supported (Radiology 2024) [225]. PCCT can distinguish dense calcium from microcalcifications [226]. A report from the PRESTIGE trial (EHJ 2024) indicated that a 1‐log rise in microcalcification burden would lead to an increased risk of long‐term ACS by 42% [227]. Therefore, “zero calcium ≠ zero risk” [228].

AROTID imaging recommends the use of percentile‐based IMT curves rather than fixed threshold values that risk overdiagnosing older people, as included in the 2022 ESH consensus [229]. ROTID imaging combines SMI, SWE, and artery brachial C‐fort (ABC), which allows the measurement of vessel structure and function simultaneously [230]. CAROTID‐ABC, a combined vascular score using SMI‐derived neovascularization, SWE‐based stiffness, and LDL‐P, was superior in predicting ASCVD events in TAILS compared with traditional IVUS [231]. Combined with ultrasmall handheld ultrasound probes and AI‐powered automatic border contouring, the new technology allowed primary care physicians to complete bilateral IMT measurements within 3 min in their practices, which offered an opportunity for decentralized service provision [232].

In the field of wearables, AI‐integrated ECGs and smartwatches allow nonstop tracking and monitoring [233]. ResNet‐WS yields an F1 of 0.94 for MIT‐BIH, indicating that an individual's 5‐year risk of developing AF is 7.6 times higher than that of other models, as measured via ResNet‐WS [234]. The Huawei WATCH D has ESH‐approved blood pressure measured by oscillometric microcuff technology, and it applies the PPG–PWTT combination algorithm on the basis of PPG technology to measure cf‐PWV under 30 s (correlation coefficient 0.89); cf‐PWV > 10 m/s predicts 2‐year MACE with a sensitivity of 85% [235].

The integration of multimodal imaging and systems biology into “imaging–electrophysiology–omics” paradigms has advanced the cutting‐edge models under evaluation [236]. The EXAM‐CV study combined CAC, SMI, AI‐ECG, and proteomics and produced a 10‐y MACE prediction C statistic of 0.93, which outperformed Framingham (0.78) and ASCVD (0.81) [237]. A federated learning architecture utilizing data from 12 hospitals spread out over different continents proved to be privacy compliant, allowed for cross‐population model testing, and still achieved an AUC decay of <0.02 [238].

## 5 Preventive Strategies and Therapeutic Interventions

**FIGURE 4:**
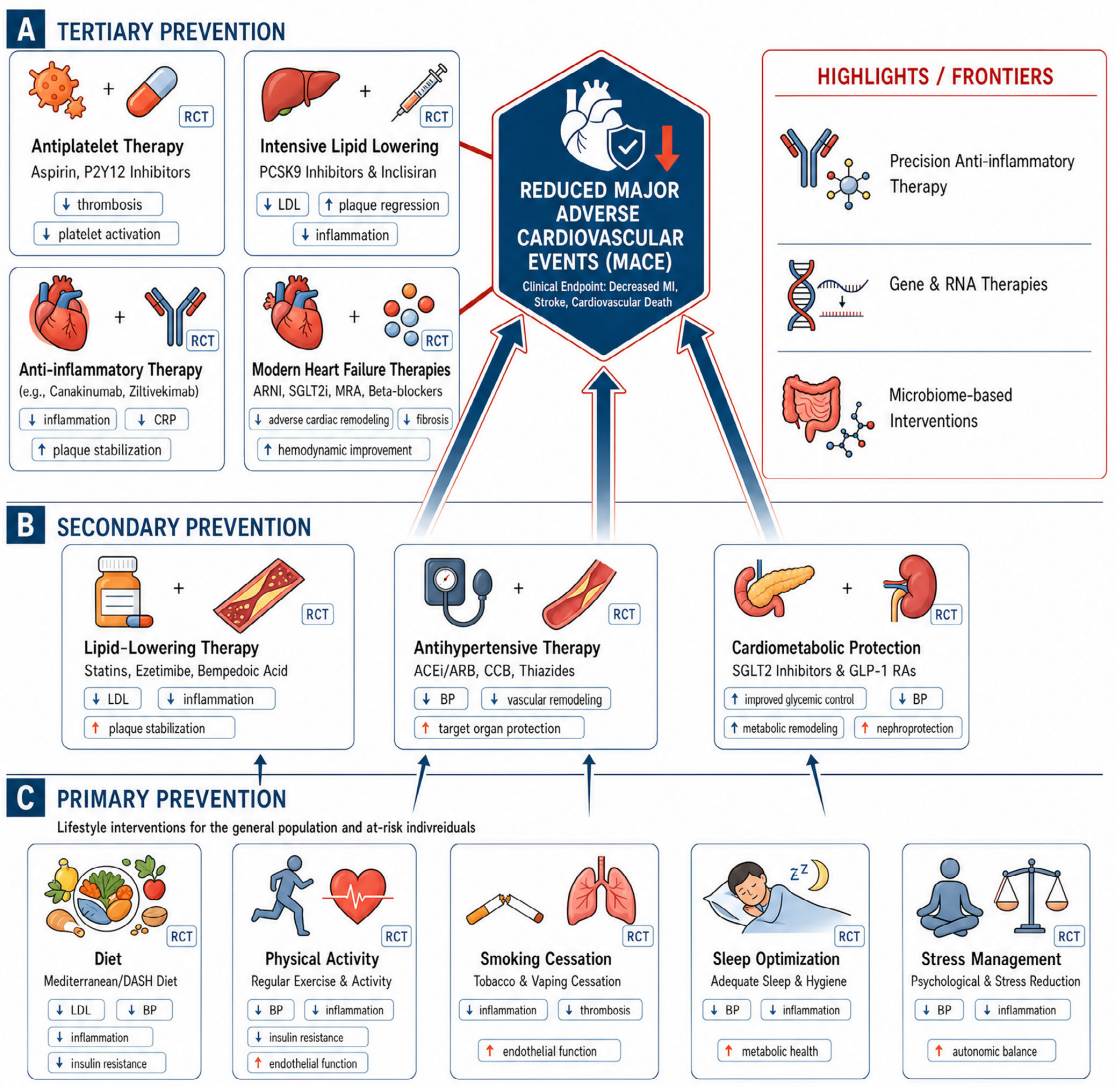
Multilayer prevention and therapeutic intervention landscape for cardiovascular disease (CVD): linking interventions to mechanisms and reduction of major adverse cardiovascular events. (Draft diagram created in WPS office PPT with GPT 5.5‐assisted layout suggestions. Final image refined and annotated by the authors. All pathway details were cross‐checked against experimental data.) (A) Lifestyle interventions (diet, exercise, smoking cessation) targeting LDL, blood pressure, and inflammation to reduce CVD risk in the general population. (B) Medication‐based management for high‐risk individuals, including statins, antihypertensives, and anti‐inflammatory strategies, to prevent disease progression. (C) Intensive management for patients with established CVD, incorporating novel approaches such as precise anti‐inflammatory treatment (NLRP3/IL‐1β/IL‐6), gene/RNA lipid‐lowering therapy (PCSK9, ANGPTL3, Lp(a)), and gut microbiome‐targeted interventions (TMAO pathway) to reduce major adverse cardiovascular events.

Preventive strategies and therapeutic interventions for CVD are increasingly shifting from a single‐factor approach to a mechanism‐informed, multilayer framework that spans primary prevention, early pharmacologic intervention for high‐risk individuals, and intensive management of established disease. Emerging evidence further highlights the context‐dependence of prevention, including gene–environment–behavior interactions and pollution‐modified treatment effects, underscoring the need for precision prevention across diverse populations. Figure 4 summarizes this tiered intervention landscape by linking lifestyle, pharmacological, and advanced therapies to mechanistic pathways and clinical outcomes while highlighting key translational frontiers such as anti‐inflammatory therapy, gene/RNA‐based lipid lowering, and microbiome‐targeted interventions.

### 5.1 Prevention Strategy

In 2022–2025, evidence from a global cohort and RCT consistently indicates that lifestyle interventions remain the cornerstone of primary prevention of CVD; however, they present a “dose–response” distribution when exposed to different contexts or pollution levels and different genotypes, which implies an emerging era of precision behavioral medicine [239, 240].

In terms of diet, a study showed that in very polluted cities where PM2.5 > 35 µg/m^3^, the lowering effect on blood pressure from a low‐sodium diet increased, and eating more foods rich in potassium could counteract, to some degree, the higher blood pressure levels caused by pollution. Subgroup analysis of the PREDIMED‐Plus trial has shown that a Mediterranean diet supplemented with nuts reduces pollution‐influenced inflammatory responses [241]. Compared with those who were not treated with interventions, there was both lower morbidity and a reduction in MACE. In terms of physical exercise, the annual increase in overall risk was 27% for people who exercised between 150 and 300 min per week at moderate intensity. However, the exercise benefit cutoff period decreased from 150 to 90 min when the PM2.5 was greater than 50 µg/m^3^ in the air. In such cases, people should follow pollution‐adjusted exercise prescriptions [241].

According to the WHO, the number of young people using e‐cigarettes on a worldwide scale has increased, and under some circumstances, there may be acute thrombotic risks [242]. The safe limit of alcohol is decreased to 0, which means that consuming any amount of alcohol contributes to the health risk of hypertension and stroke. However, air pollution RCTs have shown that wearing face masks is beneficial for public health (especially for exposed vulnerable groups) in terms of reducing the effects of harmful air pollutants. The new air quality guidelines released by the WHO in 2024 set stricter targets for the annual PM2.5 from 10 to 5 µg/m^3^, which would be able to save an additional 18.8% of CV deaths over the next 25 years [243]. From a public and policy perspective, the “9 15 Salt Reduction Week” program in China reduced population sodium intake and lowered MI and stroke incidence, highlighting the universality of some behavioral interventions for CV risk reduction. Children and pregnant women, as vulnerable populations, should receive preferential protection. Studies have shown that maternal exposure to air pollution during pregnancy increases the risk of CVD in offspring. Moreover, household air purifiers greatly improve the health outcomes of their children [244, 245].

Finally, triple interventions targeting genes, the environment, and behaviors have been proven to partially offset genetic susceptibility, lowering CVD risk among individuals with high genetic risk, indicating that the synergistic effects of precision medicine and environmental protection are taking shape.

### 5.2 Drug Therapy Interventions

For the management of hypertension, ACE inhibitors and ARBs lower blood pressure by inhibiting the RAAS system and reducing Ang II‐mediated oxidative stress and vascular smooth muscle hypertrophy. A RCT from 2022 (*n* ≈ 4800) reported that enalapril improved endothelial function, as measured by FMD, by approximately 18% compared with placebo, and that the risk of cardiovascular events in the high‐PM2.5 exposure subgroup was 11% greater [246]. Calcium channel blockers inhibit calcium influx to relax smooth muscles, which consequently alleviates vasospasm and improves coronary perfusion. Lipid management uses statins, which inhibit HMG‐CoA reductase to reduce LDL‐C to approximately 30–50% and produce pleiotropic effects, such as the upregulation of eNOS and the suppression of inflammatory mediators. The FOURIER study trials revealed that PCSK9 inhibitors (e.g., evolocumab) reduce LDL‐C by nearly 59%, and the incidence of MACE decreases by ∼15% after 3 years [247]. Air pollution can accelerate the oxidation of LDL and foam cells, and animal studies have shown that PCSK9 inhibitors can decrease the area of PM‐induced atherosclerotic plaques by ∼20%, suggesting the potential for additional benefits when there is high air pollution.

Type 2 diabetes often involves CVD, but some of the newest drugs can target both conditions—and potentially more so. SGLT2 inhibitors (e.g., empagliflozin) block the proximal tubule reabsorption of glucose and sodium and reduce hospitalization for HF by 30%. They enhance myocardial energy metabolism through increased β‐oxidation and ketone utilization. GLP‐1 receptor agonists (e.g., liraglutide) work by inhibiting inflammation via the cAMP/PKA signaling cascade and promoting endothelial repair. Liraglutide has been shown to decrease MACEs by 13%, as shown in the LEADER clinical trial [248].

Air pollution increases platelet activation via TXA_2_ synthesis and P‐selectin expression, which increases the risk of thrombosis. A multinational research study that included a total of approximately 36,000 participants reported that using aspirin regularly could lower the chance of having a MI by 27% among people who had been constantly exposed to high levels of pollution [249]. Compared with warfarin, novel oral anticoagulant drugs such as rivaroxaban can have constant antithrombotic effects, which can suffer from unstable effects due to inflammation caused by contamination. As the understanding of the mechanisms by which PM exposure causes cardiovascular damage has further expanded, there has been a shift toward drug treatment from “antipollution” to “antipollution.” Short‐term oral administration of vitamins C and E decreases the decline in FMD postexposure to high pollution by ∼45%, suggesting the use of antioxidants to prevent endothelial cell injury [250].

### 5.3 Emerging Therapeutic Interventions

Gene‐ and RNA‐based methods have progressed from the laboratory to the clinic, that is, they are shaping precision cardiovascular medicine, and the results have already shown that they are potential drivers of clinical changes. Taking single‐dose CRISPR/Cas9 as an example, gene editing reverses the clinical challenge by removing the LMNA mutation in pigs and achieves a 12% improvement in the left ventricular ejection fraction (LVEF), the first time in history that gene editing therapy has reversed HF remodeling [251]. Moreover, single‐cell sequencing revealed that p53 pathway activity decreases by 38% in cardiomyocytes, whereas apoptosis is halved compared with that in the untreated control, indicating that one‐time intervention may be beneficial in the long term [252].

PCSK9–siRNA inclisiran was found in Phase III trials (ORION‐9/10/11, *n* = 3660, 2021) to achieve an average 51% reduction in LDL‐C with a relative 25% risk reduction for MACE. Moreover, in mice fed an ApoE−/− diet and treated with a miR‐181b mimic, aorto‐coronary plaques were reduced by 42%, and plasma TNF‐α levels were decreased by 35%, demonstrating simultaneous potent dual lipid‐lowering and anti‐inflammatory effects; however, challenges remain regarding the actual delivery efficiency [253].

Lipid nanoparticles accumulate up to 70% of their dose in the liver but only three% in the heart; AAV9–CRISPR vectors optimized for cardiac targeting achieved a myocardial transduction efficiency of 45% in nonhuman primates; however, after 1 year, eight percent of off‐target edits appeared in the dorsal root ganglia [254]. The US Food and Drug Administration included “tissue‐specific promoters + inducible Cas proteins” as an example of such provisions in the industry guidance that will be published in 2024 [255]. As methods using “tissue‐specific promoters + inducible Cas proteins” reach maturity, the first multicenter gene‐editing trial in HF patients was designed to enroll 200 participants, with a primary endpoint of an 8% absolute increase in LVEF from baseline to 12 months [256].

Stem cell and immune therapies take “myocardial regeneration” from concept to real‐world effects. In the 2023 CHART‐2 multicenter, randomized trial (*n* = 240, 18‐month follow‐up), ischemic HF patients who received intracardiac injections of autologous bone marrow mesenchymal stem cells (MSCs; 1 × 106 cells/kg) presented a 5.7% increase in LVEF, an additional distance of 46 m on the 6‐min walk test and a 32% decrease in NT‐proBNP; the MRI scar mass decreased by 4.3 g, which equates to 0.02 g of scar repair per million cells injected [257]. More importantly, immune phenotypes changed, the proportion of peripheral Tregs increased from 4.1 to 6.9%, and the proportion of proinflammatory CD16+ monocytes decreased by 28%, indicating that MSCs might be capable of restoring immune homeostasis via paracrine IL‐10 and TGF‐β [257].

In the iPSC field, Japan completed the first human clinical transplantation of an autologous iPSC‐derived cardiomyocyte sheet (0.8 × 108 cells per sheet) in 2022. One year of PET perfusion imaging revealed an 18% increase in glucose uptake in the peri‐infarct myocardium and no tumorigenic signal [258]. Economic modeling with a scaled‐up production per‐course cost is projected to decrease to USD 50,000 from USD 380,000 [258]. For immunotherapy, the CANTOS extension of the IL‐1β monoclonal antibody canakinumab (*n* = 6529; 6‐year follow‐up) revealed that a high dose of the drug resulted in a 31% reduction in cardiovascular mortality, whereas an unexpected 67% reduction in the incidence of lung cancer—delivering real‐world evidence that this is truly dual anti‐inflammatory and anticancer effects [259]. In the CAR‐Treg trial launched in 2025, for the first time, antigen‐specific regulatory cells to target atherosclerotic plaques will be tested, enrolling 48 familial hypercholesterolemia subjects to measure carotid plaque volume changes as the primary endpoint [260].

### 5.4 The Current Relevant Preclinical Animal Experiments and Clinical Trials

In recent years, the treatment strategies for CVD have shifted from focusing solely on controlling individual risk factors to comprehensive prevention and treatment through multiple pathways and targets. Relevant preclinical animal experiments and clinical trials have provided abundant evidence to support this transformation. Animal experiments have shown that inflammation plays a crucial role in the development of atherosclerosis, especially the role of proinflammatory factors such as IL‐1β in the formation of arterial plaques [261]. By inhibiting these inflammatory mediators, researchers have confirmed that it is possible to effectively slow down the progression of atherosclerosis.

In terms of clinical trials, multiple large‐scale trials have verified the significance of anti‐inflammatory treatment in cardiovascular events. For instance, the CANTOS trial (NCT01327846) [262] significantly reduced the incidence of cardiovascular events by targeting the inhibition of IL‐1β, demonstrating the core role of inflammation in CVDs. Additionally, the COLCOT trial (NCT02551094) [263] further confirmed the efficacy of low‐dose colchicine in patients with MI, showing that it can effectively reduce recurrent cardiovascular events.

In terms of metabolic pathways, the efficacy of GLP‐1 receptor agonists (such as liraglutide) and SGLT2 inhibitors (such as empagliflozin) in CVDs has been widely verified in recent years. The EMPA‐REG OUTCOME trial (NCT01131676) [264] found that when used in diabetic patients, empagliflozin not only significantly reduced the mortality rate from CVDs but also decreased the incidence of HF requiring hospitalization. Similarly, the LEADER trial (NCT01179048) [265] also demonstrated that liraglutide has a significant effect in reducing MACE.

In addition to conventional drug treatment, the application of digital health and AI in CVDs is also developing rapidly. For instance, the HEARTLINE study (NCT04276441) [266] used wearable devices (such as the Apple Watch) to monitor electrocardiograms, exploring the potential of AI in the screening of AF and the prediction of cardiovascular events. The preliminary results indicated that these wearable devices have high value in monitoring cardiovascular health among high‐risk populations. Other relevant clinical trial results are summarized in Table 2.

**TABLE 2:** Representative clinical trials targeting major cardiovascular risk pathways and intervention domains.

Core pathway/intervention domain	Target or intervention	Representative trial	NCT number	Phase/study type	Status	Population/clinical setting	Evidence rationale	References
Inflammation pathway	IL‐1β inhibition with canakinumab	CANTOS	NCT01327846	Phase III	Completed; results published	Patients with prior myocardial infarction and elevated hsCRP	Landmark Phase III trial validating residual inflammatory risk	[267]
Inflammation pathway	Low‐dose colchicine	COLCOT	NCT02551094	Phase III	Completed; results published	Recent myocardial infarction	Phase III evidence; clinically applicable oral anti‐inflammatory strategy	[268]
Atherogenic lipid/LDL‐C pathway	PCSK9 inhibition with evolocumab	FOURIER	NCT01764633	Phase III	Completed; results published	Established atherosclerotic cardiovascular disease receiving statin therapy	Representative mature lipid‐lowering outcome trial	[269]
Atherogenic lipid/RNA‐based therapy	PCSK9 siRNA with inclisiran	ORION‐10/ORION‐11	NCT03399370/NCT03400800	Phase III	Completed; results published	Patients with ASCVD or ASCVD risk equivalents and elevated LDL‐C	Represents clinical maturation of RNA‐based lipid lowering	[270]
Blood pressure/vascular protection pathway	Intensive systolic blood pressure control	SPRINT	NCT01206062	Phase III	Completed; results published	High‐risk hypertensive adults without diabetes	Landmark hypertension outcome trial directly relevant to traditional risk‐factor control	[271]
Metabolic–cardiorenal pathway	SGLT2 inhibition with empagliflozin	EMPA‐REG OUTCOME	NCT01131676	Phase III	Completed; results published	Type 2 diabetes with established cardiovascular disease	Mature Phase III evidence linking diabetes, renal sodium handling, and heart failure risk	[272]
Metabolic–incretin pathway	GLP‐1 receptor agonism with liraglutide	LEADER	NCT01179048	Phase III	Completed; results published	Type 2 diabetes at high cardiovascular risk	Key GLP‐1RA cardiovascular outcome trial	[273]
Metabolic–obesity pathway	Semaglutide 2.4 mg for overweight/obesity	SELECT	NCT03574597	Phase III	Completed; results published	Overweight or obesity with established cardiovascular disease but without diabetes	Recent Phase III trial expanding CVD prevention from glycemic control to obesity treatment	[274]
Thrombosis/platelet–coagulation pathway	Low‐dose rivaroxaban plus aspirin	COMPASS	NCT01776424	Phase III	Completed; results published	Stable coronary artery disease or peripheral artery disease	Representative trial targeting residual thrombotic risk in stable ASCVD	[275]
Thrombosis/atrial fibrillation pathway	Apixaban‐based antithrombotic strategy	AUGUSTUS	NCT02415400	Phase III	Completed; results published	Atrial fibrillation with recent acute coronary syndrome and/or PCI	Clinically important trial optimizing antithrombotic balance	[276]
Digital health/AF screening pathway	Smartwatch‐based irregular pulse notification	Apple Heart Study	NCT03335800	Pragmatic digital health study	Completed; results published	General population using wearable devices	Representative large‐scale real‐world digital screening study	[277]
Digital health/rhythm monitoring pathway	Implantable loop recorder screening for AF	LOOP Study	NCT02036450	Randomized screening trial	Completed; results published	Older individuals with stroke risk factors	Important evidence for AF screening implementation and outcome uncertainty	[278]

*Abbreviations*: ASCVD, atherosclerotic cardiovascular disease; AF, atrial fibrillation; BP, blood pressure; CVD, cardiovascular disease; GLP‐1RA, glucagon‐like peptide‐1 receptor agonist; hsCRP, high‐sensitivity C‐reactive protein; LDL‐C, low‐density lipoprotein cholesterol; MACE, major adverse cardiovascular events; PCI, percutaneous coronary intervention; PCSK9, proprotein convertase subtilisin/kexin type 9; SGLT2, sodium–glucose cotransporter 2.

## 6 Challenges, Limitations, and Future Perspective

**FIGURE 5:**
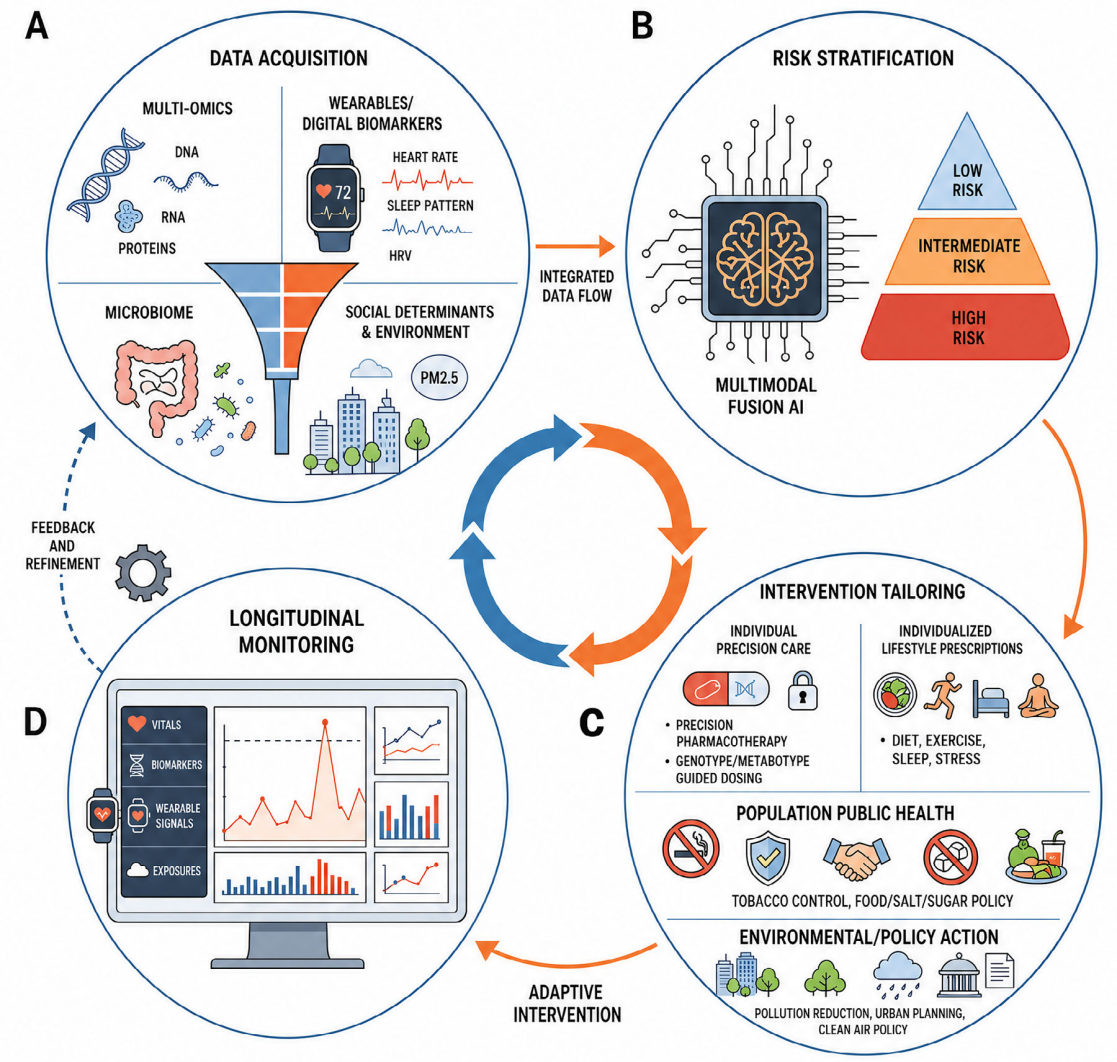
Closed‐loop systems medicine framework for future CVD management that integrates multiomics, digital biomarkers, AI, and policy actions. (Draft diagram created in WPS office PPT with GPT 5.5‐assisted layout suggestions. Final image refined and annotated by the authors. All pathway details were cross‐checked against experimental data.) (A) Multimodal data acquisition—Collection of multiomics, microbiome, wearable/digital biomarkers, and social–environmental determinants (e.g., PM2.5). (B) AI‐driven data integration—Fusion engine integrates multimodal data for refined risk stratification and individualized intervention planning. (C) Intervention strategies—Personalized care (genotype/metabotype‐guided therapy, lifestyle prescriptions), population‐level public health measures, and environmental/policy actions. (D) Longitudinal monitoring and feedback—Continuous assessment and iterative optimization of risk prediction and preventive interventions to reduce cardiovascular events.

A greater burden on the prevention and control of CVD is expected to occur under changing disease patterns, health inequity, and environmental challenges. Although enormous progress has been made using traditional approaches such as traditional therapy and public health measures, significant advances in CVD prevention and treatment are likely to come from multiomics‐based precision medicine, AI and wearable devices, prevention methods tailored for LMICs, and the mitigation of climate change, all of which could fundamentally change how CVD is prevented and treated globally (Figure 5).

Precision medicine uses genomics, transcriptomics, proteomics, and metabolomics to detect molecular mechanisms and potential therapeutic targets. In the near future, we can integrate genomic, lifestyle, and environmental‐exposure data combined with more advanced precision algorithms into an actionable tool for individualized risk assessment and treatment. AI can be combined with wearable devices, especially in electrocardiography, imaging, and medication management, to recognize risk factors efficiently, increase early prediction, and personalize interventions. The integrated applications of wearables and AI have been proven to increase the accuracy of cardiovascular event prediction. However, when analyzing from the perspective of the author in greater depth, there are still a series of systematic bottlenecks in the current research that limit the pace of this field's advancement to a higher level. In terms of the research paradigm, most existing studies are still at the stage of correlation analysis, mainly relying on cross‐sectional data or short‐term observation results, lacking longitudinal evidence of long‐term follow‐up to clarify the causal relationship between microbial group changes and the occurrence of CVDs. This “strong correlation but weak causality” research status has left many key scientific questions at the hypothesis level, especially in the mechanism between microbial metabolites (such as TMAO) and atherosclerosis, lacking direct intervention evidence support. From the technical perspective, significant differences in sample collection (such as feces, blood or oral samples), sequencing strategies (16S rRNA sequencing and metagenomic sequencing), and bioinformatics analysis processes among different studies lead to high heterogeneity in research results. This methodological inconsistency not only weakens the reproducibility of research results but also poses a significant challenge to cross‐study integration analysis. Moreover, the microbial group itself has high individual specificity and is dynamically influenced by multiple factors such as dietary structure, drug use (such as antibiotics), lifestyle, and geographical environment. This complexity significantly limits the extrapolation of research results in different populations and also increases the difficulty of establishing a unified biomarker system. More critically, although a large number of studies in recent years have proposed potential molecular mechanisms and intervention targets, the studies that have truly entered the clinical validation stage are still limited. There are also no high‐quality RCTs to evaluate the effectiveness and safety of microbial intervention strategies, which largely restricts the transformation process of this field from basic research to clinical application.

This review has several limitations. First, although we aimed to provide a comprehensive overview of traditional and emerging CVD risk factors, screening strategies, preventive approaches, and therapeutic interventions, the article is narrative in nature rather than a formal systematic review or meta‐analysis; therefore, study selection, evidence weighting, and interpretation may be influenced by publication availability and author judgment. Second, the included evidence spans diverse populations, study designs, geographic regions, and socioeconomic settings, resulting in substantial heterogeneity in exposure definitions, outcome measurements, follow‐up duration, and adjustment for confounders, which limits direct comparison across studies. Third, for several emerging risk factors—such as gut microbiota, noncoding RNAs, microplastics/nanoparticles, psychosocial stress, climate‐related exposures, and multiomics biomarkers—current evidence remains largely observational or preclinical, and causal relationships, standardized measurement methods, clinically actionable thresholds, and long‐term intervention benefits have not been fully established. Fourth, many advanced screening and prediction tools, including AI‐enhanced electrocardiography, wearable devices, multimodal imaging, polygenic risk scores, and multiomics‐based models, have shown promising performance in selected cohorts but still require external validation across ancestries, age groups, sex‐specific populations, and LMICs before broad clinical implementation. Fifth, data from resource‐limited settings remain insufficient, particularly regarding longitudinal outcomes, cost‐effectiveness, treatment adherence, environmental exposure assessment, and real‐world feasibility, which may reduce the generalizability of some recommendations to populations with the highest CVD burden. Finally, the translation of precision cardiovascular medicine into routine practice faces unresolved challenges, including data privacy, algorithmic bias, interoperability of digital health systems, regulatory oversight, affordability, clinician acceptance, and equitable access. Future studies should prioritize large‐scale prospective cohorts, RCTs, standardized biomarker and exposure assessment protocols, and implementation research in diverse healthcare systems to strengthen causal inference and support scalable, equitable CVD prevention and management.

Future research should vigorously promote large‐scale, multicenter, and prospective cohort studies. Through long‐term dynamic monitoring of microbial group changes and combined with clinical outcome data, the causal relationship between the microbiome and CVDs as well as their temporal sequence characteristics can be more accurately analyzed. At the same time, conducting gene–environment–microbial interaction studies based on genetic information will help reveal the biological basis of individual differences. In terms of technology and methods, integrating multiomics data (including metagenomics, metabolomics, transcriptomics, and proteomics) and introducing AI and ML algorithms are expected to construct more precise disease prediction models and complex regulatory networks, thereby identifying key drivers and potential intervention targets. Finally, in the translational application aspect, strengthening intervention research on the microbiome, such as strategies based on probiotics, dietary regulation, fecal microbiota transplantation, and small molecule drugs targeting metabolic pathways, and systematically evaluating their efficacy and safety through well‐designed randomized controlled clinical trials, will promote this field from theoretical exploration to precise medical practice. LMICs remain the dominant contributors to total CV death, which is above 45%. Owing to their lack of medical infrastructure as well as public health and administrative resources, any improvements that can be made should include policies, awareness programs, education about community management, early detection, and high‐risk group intervention. Climate change increases the likelihood of CVD. Higher levels of pollution, more severe heat waves, and worse storms increase the risk of more CVD and higher death rates. It was recommended that governments should both increase investments in and improve air quality and increase their ability to respond efficiently in emergency cases. With policies, interconnections, sectors, and science working together, proper implementation can prevent more illnesses from continuing to stem from climate change, improve cardiovascular prevention, and promote the development of more solutions to help everyone involved. The main content of this article is summarized in Table 3.

**TABLE 3:** Summary of cardiovascular disease risk factors, screening tools, preventive strategies, and therapeutic interventions.

Category	Key factors/tools	Core biological mechanisms (core pathways)	Clinical relevance/evidence	Screening/monitoring	Prevention and therapeutic interventions	References
Global CVD burden and epidemiology	Global/regional distribution; LMIC vs. HIC trends; China‐specific burden	Lifetime risk accumulation; demographic transition; urbanization	CVD mortality rising globally; marked regional heterogeneity	National registries; GBD/WHO datasets	Population prevention + health‐system strengthening	[14, 15, 16, 17, 18], [34, 35, 36, 37, 38, 39, 40, 41, 42, 43, 44, 45, 46, 47, 48, 49, 50, 51]
Traditional risk factors	Hypertension, diabetes, dyslipidemia, smoking, obesity, sedentary lifestyle	Endothelial dysfunction; oxidative stress; VSMC phenotypic switching; insulin resistance	Major attributable fraction; residual risk remains despite control	BP, HbA1c, lipid panel, BMI	Lifestyle + guideline‐directed therapy	[1, 2, 3, 4, 5, 6, 7, 8, 9, 10, 11, 12], [93, 94, 95, 96, 97, 98, 99, 100, 101, 102, 103, 104, 105, 106, 107, 108, 109, 110]
Lipid‐related residual risk	Lp(a), TG‐rich remnants (VLDL/IDL), LDL particle number	Atherogenic lipoproteins; inflammation cross‐talk	Independent risk; contributes to residual risk poststatin	Lp(a), ApoB, LDL‐P	PCSK9/LPA‐targeted strategies; combination lipid lowering	[103, 104, 105], [145]
Environmental factors	PM2.5/NO_2_, noise, heat waves, climate change	Oxidative stress; autonomic imbalance; endothelial injury	8–18% mortality risk increase per PM2.5 increment; heat‐wave mortality	Exposure modeling; population‐level monitoring	Policy mitigation; exposure reduction; pollution‐adjusted prescriptions	[13], [117], [243, 244, 245]
Psychosocial and social determinants	Chronic stress, depression, social isolation, unemployment shocks	HPA‐axis activation; IL‐6/CRP inflammation; autonomic dysregulation	Dose–response harm; drives disparities	Psychometric tools; HRV; social risk screening	CBT/mindfulness; community interventions; policy support	[118, 119, 120, 121, 122, 123, 124, 125, 126]
Inflammation and infection	Chronic low‐grade inflammation; periodontitis; viral infections (incl. SARS‐CoV‐2)	Plaque vulnerability; immune activation	Anti‐inflammatory therapy reduces events; postinfectious CV events	hsCRP; inflammatory biomarkers	Anti‐inflammatory modulation; vaccination and early postinfection management	[105], [127], [137, 138]
Genetics and epigenetics	Family history; GWAS loci; SNPs (e.g., 9p21); noncoding RNAs (miRNAs/lncRNAs)	Gene–environment interaction; epigenetic regulation; miRNA‐mediated remodeling	Improves individualized risk assessment; enables targeted therapies	PRS/GWAS; circulating miRNA panels	Precision medicine; RNA‐targeted strategies	[71, 139, 140, 141, 142, 143, 144, 145, 146, 147, 148, 149, 150, 151, 152, 153]
Gut microbiota/toxins	Gut microbiota; nanoparticle/toxin exposure; indole metabolites	Th17/Treg balance; SCFAs; immune–metabolic signaling	Emerging evidence	Metabolomics/microbiome sequencing (research)	Diet‐based modulation; microbiome‐targeted therapy (future)	[110], [154, 155, 156, 157, 158, 159]
Clinical risk scoring	Framingham/SCORE; region‐specific models (e.g., China‐PAR, CKB, 4C)	Multifactor aggregation; lifetime exposure	Widely used; limited for emerging risks	Routine labs + demographics	Risk‐guided primary prevention	[95], [204], [206]
Imaging‐based screening	CAC progression; CCTA; carotid ultrasound (IMT/SMI/SWE); PCCT	Subclinical atherosclerosis detection; microcalcification burden	CAC progression predicts MACE; guideline incorporation	CAC/CCTA; carotid IMT; advanced plaque imaging	Early initiation/intensification of prevention	[214, 215, 216, 217, 218, 219, 220, 221, 222, 223, 224, 225, 226, 227, 228, 229, 230]
Biomarkers and molecular screening	LDL‐P, hsCRP, multiomics biomarkers	Mechanism‐linked risk stratification	Evolving evidence	Proteomics/metabolomics panels	Biomarker‐guided treatment intensification	[221, 222, 223, 224, 225, 226, 227, 228, 229, 230]
AI and digital health	AI‐enabled ECG; smartwatches; PWV‐based BP prediction; wearable monitoring	Multimodal integration; early detection	Increasing validation; improving prediction	Continuous ECG/BP/HRV	Digital coaching + risk prediction + adherence support	[218, 219, 220]
Lifestyle and behavioral prevention	Mediterranean diet; exercise prescriptions adjusted by pollution; smoking/alcohol control	Anti‐inflammatory; endothelial protection; metabolic benefit	Strong RCT/cohort evidence; gene–environment interactions	Wearables; adherence monitoring	Precision behavioral medicine; policy campaigns	[239, 240, 241, 242, 243, 244, 245]
Drug therapy interventions	ACEI/ARB, CCB; lipid‐lowering; glucose‐lowering; anti‐inflammatory agents	RAAS modulation; LDL lowering; glucose excursion stabilization; inflammation targeting	RCT evidence; subgroup effects by environment/genotype	BP/lipids/HbA1c + imaging follow‐up	Multitarget combination and precision therapy	[105, 106, 107], [246]
Future perspectives	Multiomics precision medicine; AI + wearables; LMIC‐tailored programs; climate mitigation; CAR‐Treg trial	Closed‐loop prevention: data→risk→intervention→feedback	High potential but needs implementation	Integrated platforms (omics + her + environment)	Precision prevention + policy integration	[260]

*Abbreviations*: LMIC: low‐ and middle‐income countries; HIC: high‐income countries; GBD: Global Burden of Disease; WHO: World Health Organization; VSMC: vascular smooth muscle cell; BP: blood pressure; HbA1c: glycated hemoglobin A1c; BMI: body mass index; Lp(a): lipoprotein(a); TG: triglyceride; VLDL: very‐low‐density lipoprotein; IDL: intermediate‐density lipoprotein; LDL: low‐density lipoprotein; ApoB: apolipoprotein B; LDL‐P: low‐density lipoprotein particle number; PCSK9: proprotein convertase subtilisin/kexin type 9; LPA: lipoprotein(a) gene; PM2.5: particulate matter ≤ 2.5 µm; NO_2_: nitrogen dioxide; HPA: hypothalamic–pituitary–adrenal; IL‐6: interleukin‐6; CRP: C‐reactive protein; HRV: heart rate variability; CBT: cognitive behavioral therapy; SARS‐CoV‐2: severe acute respiratory syndrome coronavirus 2; CV: cardiovascular; hsCRP: high‐sensitivity C‐reactive protein; GWAS: genome‐wide association study; SNP: single‐nucleotide polymorphism; miRNA: microRNA; lncRNA: long noncoding RNA; PRS: polygenic risk score; Th17: T helper 17 cell; Treg: regulatory T cell; SCFA: short‐chain fatty acid; SCORE: Systematic Coronary Risk Evaluation; China‐PAR: China Prediction for Atherosclerotic Cardiovascular Disease Risk; CKB: China Kadoorie Biobank; CAC: coronary artery calcium; CCTA: coronary computed tomography angiography; IMT: intima–media thickness; SMI: superb microvascular imaging; SWE: shear wave elastography; PCCT: photon‐counting computed tomography; MACE: major adverse cardiovascular events; AI: artificial intelligence; ECG: electrocardiogram; PWV: pulse wave velocity; RCT: randomized controlled trial; ACEI: angiotensin‐converting enzyme inhibitor; ARB: angiotensin receptor blocker; CCB: calcium channel blocker; RAAS: renin–angiotensin–aldosterone system; EHR: electronic health record; CAR‐Treg: chimeric antigen receptor regulatory T cell.

## Author Contributions

ZZ and MK wrote the manuscript. MH and XZ reviewed and SH revised the manuscript. YY and CZ provided critical revisions. All authors have read and approved the final manuscript.

## Funding

This work was supported by the National Natural Science Foundation of China (Grant Nos. U23A20398 and 82030007) and the Noncommunicable Chronic Diseases‐National Science and Technology Major Project (Grant No. 2024ZD0537707).

## Ethics Statement

The authors have nothing to report.

## Conflicts of Interest

The authors declare no conflicts of interest.

## Data Availability

The authors have nothing to report.
